# On-Resin Recognition of Aromatic Oligopeptides and Proteins through Host-Enhanced Heterodimerization

**DOI:** 10.1021/jacs.2c02287

**Published:** 2022-05-10

**Authors:** Xiaoyi Chen, Zehuan Huang, Renata L. Sala, Alan M. McLean, Guanglu Wu, Kamil Sokołowski, Katie King, Jade A. McCune, Oren A. Scherman

**Affiliations:** † Melville Laboratory for Polymer Synthesis, Yusuf Hamied Department of Chemistry, University of Cambridge, Cambridge CB2 1EW, U.K.

## Abstract

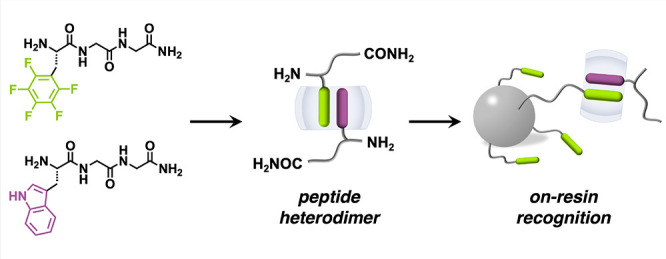

Peptide dimerization is ubiquitous in natural protein conjugates and artificial self-assemblies. A major challenge in artificial systems remains achieving quantitative peptide heterodimerization, critical for next-generation biomolecular purification and formulation of therapeutics. Here, we employ a synthetic host to simultaneously encapsulate an aromatic and a noncanonical l-perfluorophenylalanine-containing peptide through embedded polar−π interactions, constructing an unprecedented series of heteropeptide dimers. To demonstrate the utility, this heteropeptide dimerization strategy was applied toward on-resin recognition of *N*-terminal aromatic residues in peptides as well as insulin, both exhibiting high recycling efficiency (>95%). This research unveils a generic approach to exploit quantitative heteropeptide dimers for the design of supramolecular (bio)systems.

**P**eptide dimerization through either covalent or noncovalent bonding is key in structural design and functional control of natural^1^ and artificial^2^ self-assembly. Covalent conjugation requires elaborate reactions to form static, strong covalent bonds between peptides,^3^ while noncovalent peptide dimerization is facile and versatile,^4^ on account of its dynamic and reversible nature. However, the relatively weak association and low specificity have limited its use in aqueous systems. To address this problem, various synthetic hosts have been utilized to encapsulate hydrophobic peptide residues within their nanocavities, enhancing the overall binding strength of noncovalent peptide dimers.^5^

On account of their high binding affinity and range of guests, cucurbit[*n*]uril (CB[*n*]) macrocyclic hosts are ideal to bind peptides.^6−11^ Urbach and co-workers reported a homopeptide dimer between two FGG tripeptides and CB[8], displaying high binding strength (*K* ≈ 10^11^ M^–2^).^6^ This homodimer has been adopted as a versatile building block in the design and fabrication of supramolecular oligomers,^12^ polymers,^13,14^ hydrogels,^15,16^ and protein/peptide assemblies.^17^ Although significant advances have been made, a major challenge remains favorable, quantitative formation of heteropeptide dimers without homodimerization.

Herein, we employ CB[8] to mediate heterodimerization of a canonical aromatic peptide and a noncanonical l-perfluorophenylalanine(F′)-containing peptide, Figure 1a. Recently, we reported that an electron-poor perfluorophenyl first guest and an electron-rich phenyl second guest can exclusively form a CB[8]-mediated heteroternary complex through host-enhanced polar−π interactions.^18,19^ Thus, we postulated that the F′-containing peptide (F′GG) would exclusively form a 1:1 complex with CB[8], avoiding homodimerization on account of the electrostatic repulsion within a 2:1 complex. Subsequent association of various aromatic peptides (e.g., WGG) with the F′GG-CB[8] complex may enable access to a new host-enhanced heteropeptide dimer with superior binding strength, Figure 1a.

**Figure 1:**
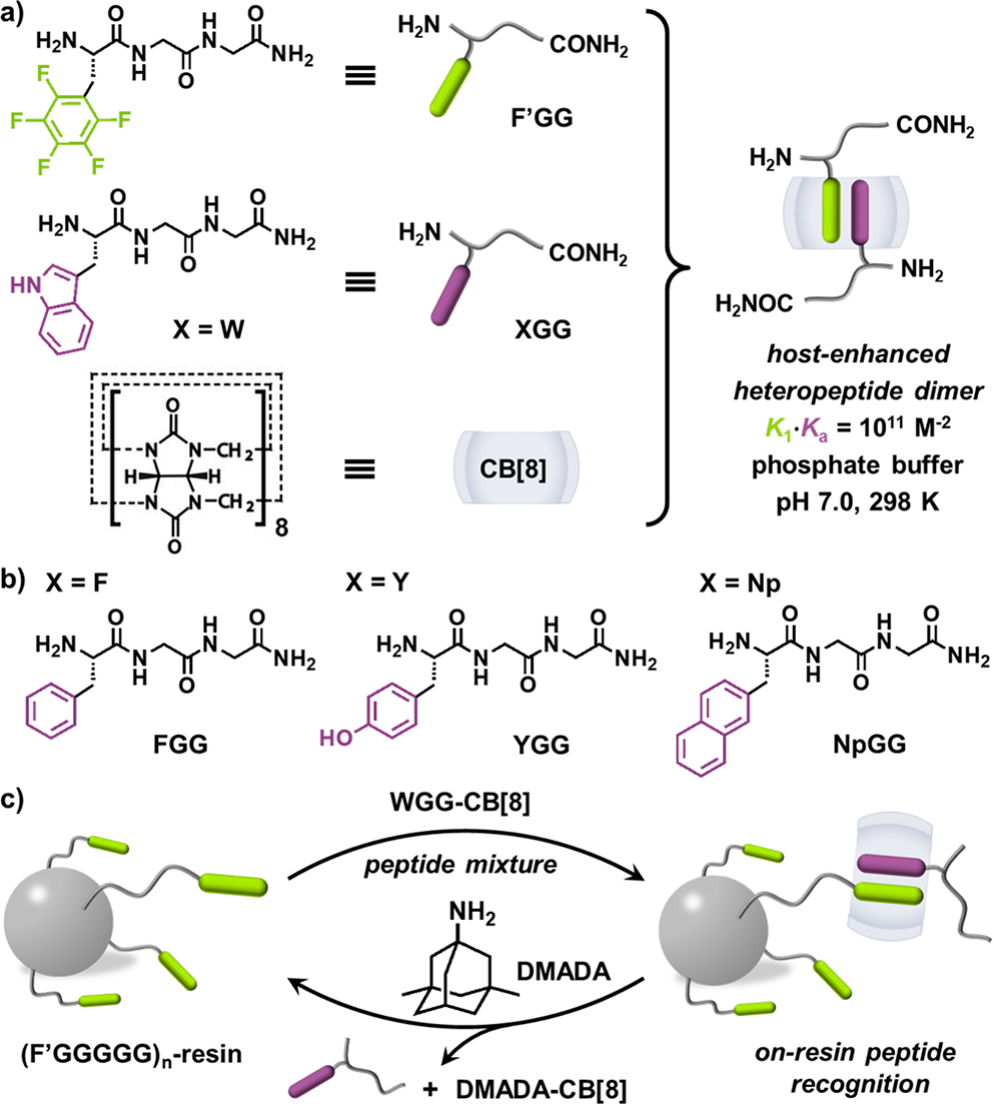
Schematic of (a) host-enhanced heteropeptide dimer formed from F′GG, WGG, and CB[8]; (b) molecular structures of FGG, YGG, NpGG; (c) on-resin recognition through interfacial heterodimerization.

Five model aromatic tripeptides containing l-perfluorophenylalanine^20^ (F′GG), L-tryptophan (WGG), l-phenylalanine (FGG), l-tyrosine (YGG), and l-(2-naphthyl)alanine (NpGG) at the N-termini were designed and prepared, Figure 1a–b. An equimolar mixture of F′GG, CB[8], and **X**GG should result in an exclusive heterodimer instead of an equilibrium mixture containing homodimers. Two additional series of tripeptides (GG**X**, G**X**G), containing aromatic amino acids either at the C-termini or in the midchain, were synthesized to investigate a range of second guests and the effect of their position in the oligopeptides, Chart S1. After elucidating binding thermodynamics, we applied this heterodimerization to achieve *on-resin* recognition and isolation^21,22^ of aromatic tripeptides from a peptide mixture exhibiting high efficiency and selectivity, Figure 1c.

^1^H and ^19^F NMR titrations were performed to probe heteropeptide dimerization within CB[8], Figure 2a and Figures S1–S16. Titration of WGG into a 1:1 mixture of F′GG-CB[8] resulted in a gradual appearance of indole protons at 6.25–7.10 ppm. On account of shielding from the CB[8] cavity, these proton peaks exhibited upfield shifts compared to free WGG, suggesting that the indole group of F′GG-CB[8]-WGG is located in a different chemical environment from unbound WGG. This titration was also monitored by ^19^F NMR, Figure 2a; a new group of fluorine peaks gradually appeared, while the peaks of F′GG-CB[8] disappeared. Additionally, equivalent mixtures of F′GG, CB[8], and XGG were characterized by high-resolution ESI-MS, Figure 2b. All ion peaks for F′GG-CB[8]-WGG, F′GG-CB[8]-FGG, F′GG-CB[8]-YGG, and F′GG-CB[8]-NpGG complexes were identified at their calculated *m*/*z* values. Together, these data confirmed the successful formation of new heteropeptide dimers.

**Figure 2:**
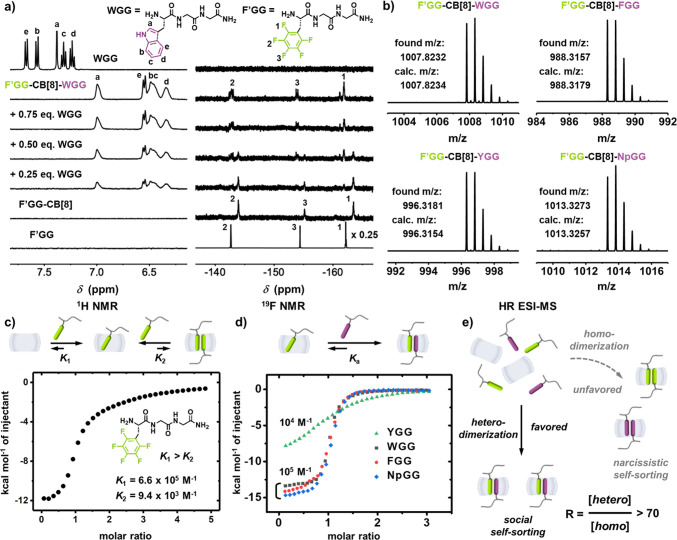
(a) ^1^H and ^19^F NMR spectra (D_2_O, 298 K) obtained through titration of WGG (16.0 mM) into F′GG-CB[8] (1.0 mM); (b) HR ESI-MS spectra of heteropeptide dimers (H_2_O, 1.0 mM) of WGG, FGG, YGG, NpGG with 1:1 complex of F′GG-CB[8]; ITC titration plots (10 mM phosphate buffer, pH 7.0, 298 K) of (c) F′GG (3.0 mM) into CB[8] (0.1 mM); (d) WGG, FGG, YGG, NpGG (3.0 mM) into F′GG-CB[8] (0.2 mM); and (e) schematic of self-sorting mechanism.

Isothermal titration calorimetry (ITC) was employed to study binding thermodynamics of heteropeptide dimerization (Figure 2c and Figure S21–S25). Titration of F′GG (3.0 mM) into CB[8] (0.1 mM) led to a stepwise binding curve with two transitions at molar ratios of 1.0 and 2.0 (Figure 2c). The first-step binding constant (*K*_1_) is 6.6 × 10^5^ M^–1^, while the second-step (*K*_2_) is 9.4 × 10^3^ M^–1^. This indicates negative cooperativity where *K*_1_ > *K*_2_.^18,19,23^ Such favorable 1:1 complexation enables secondary access of electron-rich aromatic peptides. Titrations of XGG (X = W, F, Y, Np; 3.0 mM) into F′GG-CB[8] (0.2 mM) resulted in four binding curves with a clear transition at 1.0 molar ratio (Figure 2d), indicating successful incorporation of XGG into F′GG-CB[8].

Table 1 shows that all XGG peptides exhibited high binding strengths (*K*_a_ > 10^4^ M^–1^), confirming thermodynamic stability of heteropeptide dimerization. YGG (Figure 2d, green) displayed a relatively low *K*_a_, as the para-substituted hydroxyl group may decrease enthalpic contributions, weakening the second association.^24^ Nevertheless, the overall binding constants (*K*_1_·*K*_a_) for the heteropeptide dimers F′GG-CB[8]-XGG are all higher than 10^10^ M^–2^. This shows significant enhancement compared to their parent dimers (e.g., F′GG-WGG,^25,26^*K*_dimer_ ≈ 1 M^–1^). The overall *K*_1_·*K*_a_ for F′GG-CB[8]-FGG (heterodimer 2.4 × 10^11^ M^–2^) is higher than that for 2FGG-CB[8] (homodimer, 1.5 × 10^11^ M^–2^),^6^ on account of the enhanced polar−π interactions. Notably, no secondary association of nonaromatic analogs (KGG, EGG, LGG) was observed, highlighting selectivity for aromatic over nonaromatic peptides.

**Table 1:** Thermodynamic Data for Secondary Association of Aromatic Residue-Containing Tripeptides and Control Tripeptides with the 1:1 Complex of F′GG-CB[8]^a^

model peptide	*K*_a_ (10^3^ M^–1^)	*ΔH*_a_ (kcal mol^–1^)	–*TΔS*_a_ (kcal mol^–1^)
**W**GG	460 ± 53	–13.3 ± 0.2	5.6 ± 0.2
G**W**G	103 ± 6	–11.9 ± 0.1	5.0 ± 0.1
GG**W**	90 ± 7	–14.5 ± 0.3	7.8 ± 0.4
**F**GG	356 ± 26	–14.4 ± 0.4	6.8 ± 0.3
G**F**G	58 ± 3	–9.1 ± 0.4	2.6 ± 0.4
GG**F**	22 ± 1	–7.5 ± 0.2	1.5 ± 0.2
**Y**GG	3 ± 5	–9.6 ± 0.1	3.6 ± 0.2
G**Y**G	4 ± 0.3	–4.7 ± 0.2	–0.2 ± 0.3
GG**Y**	–b	–b	–b
**Np**GG	382 ± 22	–14.6 ± 0.2	7.0 ± 0.2
G**Np**G	165 ± 10	–12.7 ± 0.4	5.6 ± 0.4
GG**Np**	90 ± 2	–12.0 ± 0.1	5.2 ± 0.1
KGG	–b	–b	–b
EGG	–b	–b	–b
LGG	–b	–b	–b

a Averaged with three replicates.

b Not detected.

To understand the influence of aromatic position on heterodimerization, *K*_a_ values of GXG and GGX (X = W, F, Y, Np) with F′GG-CB[8] were determined by ITC. Shifting aromatic residues from N- to C-termini led to a notable decrease in *K*_a_, Table 1. The increased distance between the positive charge at the N-terminus and the aromatic motif weakens ion–dipole interactions at the CB[8] portal, reducing the secondary binding affinity. This is exemplified by GGY, where no secondary binding to F′GG-CB[8] was observed. Seven new, derivatized heteropeptide dimers, F′GG-CB[8]-GXG and F′GG-CB[8]-GGX, expand the scope of host-enhanced heteropeptide dimerization. The exhibited binding selectivity to aromatic residues is an advantage of this system, enabling access to a range of peptides and proteins. Compared to previous reports on CB[8]-peptide heteroternary complexes,^27^ the system described here is simply based upon an F′ amino acid, easily accessible for ligation in chemical biology and biochemistry.

The thermodynamic mechanism behind peptide heterodimerization is attributed to social self-sorting, Figure 2e, consistent with previous reports.^18,19^ Two pathways exist in an equimolar mixture of F′GG, CB[8], and XGG: social self-sorting (heterodimerization) and narcissistic self-sorting (homodimerization). Compared to homodimerization (Table S1), social self-sorting was favored in the presence of a ratio of hetero- and homopeptide dimers (e.g., WGG, R = 2291), confirming quantitative heteropeptide dimerization in the complex mixture. Notably, simply mixing two aromatic tripeptides with CB[8] does not lead to quantitative heterodimerization (Figures S17–S19).

After elucidation of thermodynamics, we demonstrated the utility of heteropeptide dimers to achieve on-resin recognition of aromatic peptides, Figure 1c. Confocal fluorescence imaging was employed to probe interfacial recognition through a fluorescent tagged peptide (WGGGGG-dansyl),^9^Figure 3a–b. A buffered solution of WGGGGG-dansyl-CB[8] (10 mM) was mixed with F′GGGGG-functionalized ChemMatrix resin (35–100 mesh particle size, surface loading = 0.5 mmol/g) for 10 min at 25 °C with vigorous shaking. Confocal fluorescent images were captured under a gray field upon laser irradiation (λ = 405 nm). Figure 3c–f show that the resin with F′GGGGG-CB[8]-WGGGGG-dansyl displays significantly higher fluorescence than F′GGGGG, F′GGGGG-CB[8]-WGG, and F′GGGGG-WGGGGG-dansyl (Tables S3–S4), indicating the successful heteropeptide dimerization on resin.

**Figure 3:**
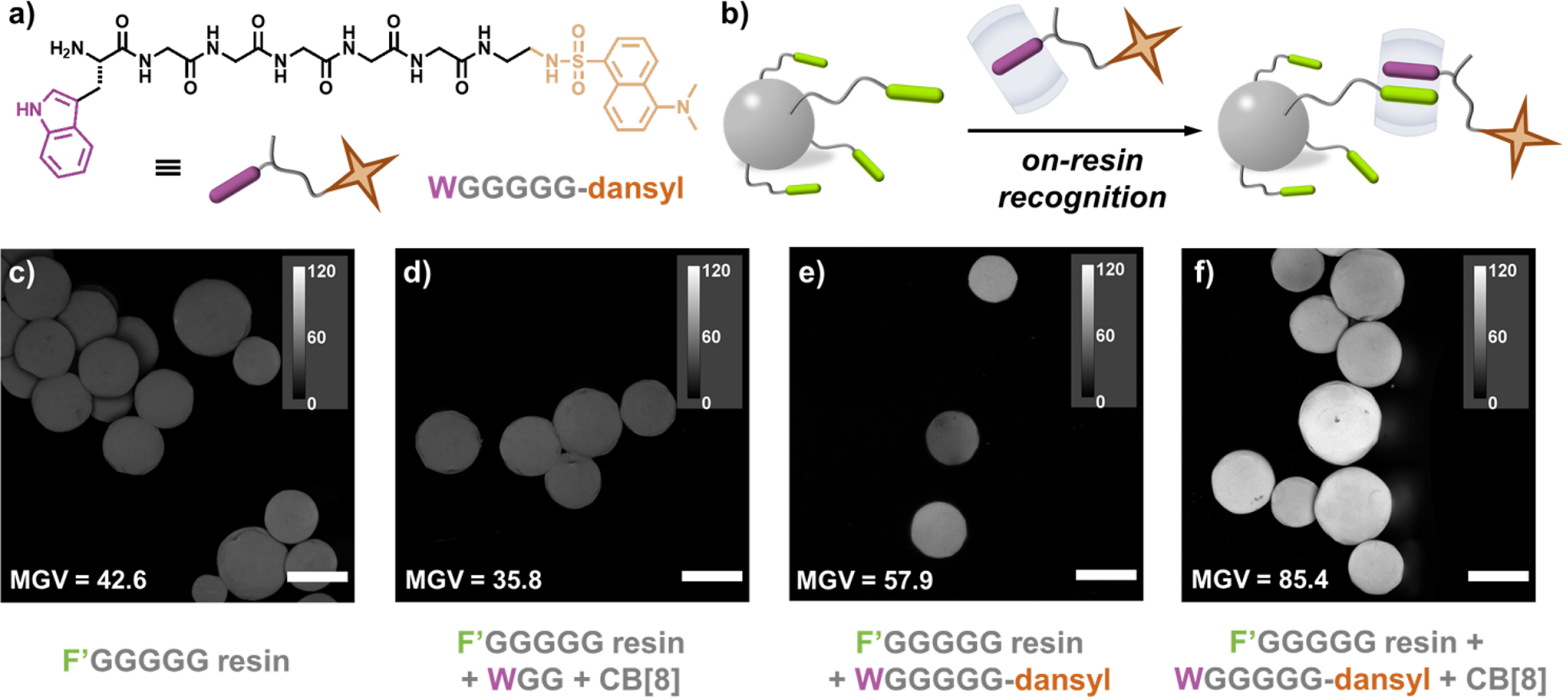
Schematic of (a) molecular structure of WGGGGG-dansyl, and (b) interfacial recognition with F′GGGGG resin (10 mM); confocal fluorescent images of (c) F′GGGGG resin, and those added with (d) WGG-CB[8] (10 mM), (e) WGGGGG-dansyl (10 mM), and (f) WGGGGG-dansyl-CB[8] (10 mM) obtained through laser excitation at λ = 405 nm under a gray field. Scale bar = 200 μm. Fluorescence intensity is quantified by mean gray value (MGV).

UV experiments were performed to test absorption efficiency through quantification of aromatic peptides present before and after on-resin treatment, Figure 4a. A typical experiment involved mixing WGG-CB[8] (1.0 mM) with F′GGGGG-resin (10.0 mM) at 25 °C for 10 min. The absorption intensity of the resin-treated WGG-CB[8] (gray) showed a decrease compared to the original (purple), Figure 4b. The absorption efficiency for on-resin recognition was 77%, while recognition of free WGG by physical absorption was only 19%, Table S5. Absorbed WGG was released and recycled through competitive binding by memantine hydrochloride (DMADA), Figure S29, regenerating the resin.

**Figure 4:**
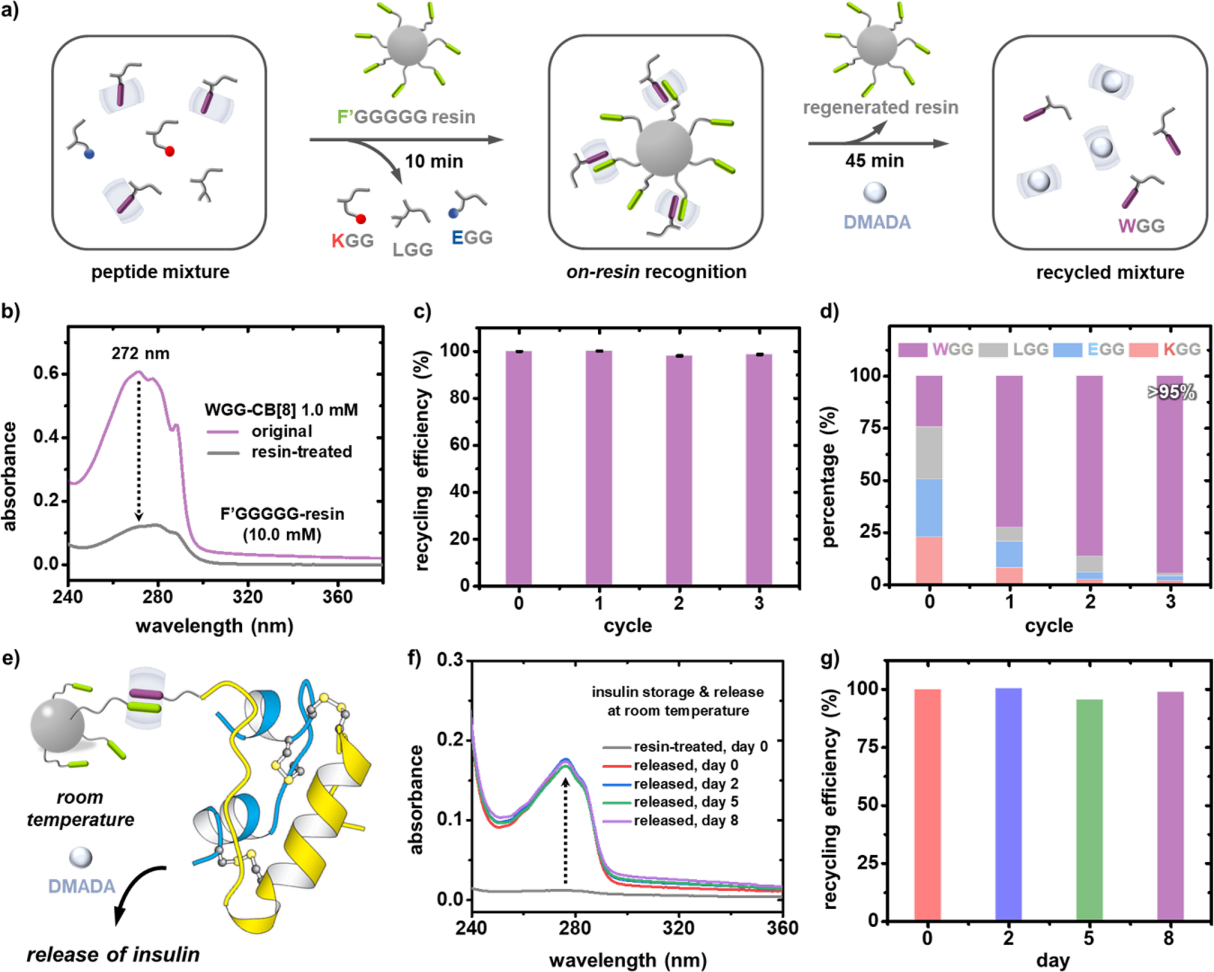
(a) Schematic of on-resin recognition and separation of an aromatic tripeptide from a peptide mixture; (b) UV spectra of WGG-CB[8] (1.0 mM) before and after treatment with F′GGGGG-resin (10.0 mM); histograms of (c) recycling efficiency of WGG in continuous on-resin recognition cycles; (d) percentage of WGG obtained after multicycle isolation; (e) schematic of on-resin stabilization of insulin, on-demand release and binding to CB[8], not drawn to scale; (f) UV spectra of insulin (0.2 mM) before and after treatment with F′GGGGG-CB[8]-resin (10.0 mM) followed by release at days 0, 2, 5, and 8; (g) histograms of recycling efficiency of insulin at days 0, 2, 5, and 8.

Multicycle on-resin recognition was performed to evaluate recyclability, Figure 4c and Table S6. Recognition-regeneration experiments on WGG-CB[8] were repeated for 3 cycles using the same batch of resin. The on-resin recycling efficiency was maintained above 98% over multiple cycles (Figure 4c), on account of complete release of WGG without any residue accumulation. This confirms regeneration of F′GGGGG-functionalized resin, endowing the whole process with high sustainability for practical use. We further investigated selective isolation of aromatic peptides through recognition-release experiments over 3 cycles (Figure 4d and Table S7), using a peptide mixture of WGG, KGG, EGG, and LGG ([XGG] = 1.0 mM) in the presence of 1.0 mM CB[8]. The ratio of WGG (purple) within the mixture is increased from 26 to >95% after 3 cycles (Figure 4d). Additionally, residual DMADA and DMADA-CB[8] can be removed through liquid-phase chromatography. This facile strategy to obtain aromatic peptides with high purity through on-resin heteropeptide dimerization is readily amenable to automation.

To extend applicability of this approach, we exploited interfacial recognition for insulin stabilization and its on-demand release from the resin, Figure 4e and Table S8. Insulin is a widely used biopharmaceutical for diabetes treatment;^28^ however, on account of limited stability it requires strict storage conditions (e.g., 2–6 °C) as it is prone to form immunogenic fibrillar aggregates in solution.^29^ Insulin has an N-terminal phenylalanine, which can serve as a guest for CB[8].^30−32^ Heteropeptide dimerization of insulin and F′GGGGG-functionalized resin may offer a promising solution to address insulin instability.

UV experiments quantified insulin absorbance onto the resin and on-demand release. Insulin absorption efficiency was calculated to be 94%, and its absorption intensity decreased after treatment with F′GGGGG-CB[8] resin, Figure 4f and Figure S34. Through competitive binding, insulin was displaced by DMADA with ∼95% recycling efficiency (Figure 4g) over 8 days of storage at room temperature, indicating long-term stability (Figure S35 and Table S9). Our approach provides a route for storing insulin under ambient conditions, removing the current need for refrigeration.

In conclusion, we have introduced a new type of quantitative heteropeptide dimerization. Through host-enhanced polar−π interactions, the binding affinity between aryl and perfluorophenyl groups from two different peptides is significantly enhanced with a *K*_a_ up to 10^5^ M^–1^ and a *K*_1_·*K*_a_ up to 10^11^ M^–2^, ensuring exclusive formation of heteropeptide dimers. To demonstrate utility, the solution-phase host–guest complex (F′GG-CB[8]-XGG) was transferred to a solid–liquid interface achieving on-resin recognition and isolation of aromatic peptides as well as stabilization and on-demand release of insulin under ambient conditions. This generic approach enables accumulation and separation of aromatic-abundant biomacromolecules useful in biomedical research. We anticipate that this work will inspire research into exploitation of heteropeptide dimerization as a versatile strategy for a wide range of life science applications.

## Supporting Information Available

Materials and instrumentation, synthesis and characterization of model peptides, NMR spectra of all heteropeptide dimers, ITC titration plots of peptide heterodimerization, statistical analysis of confocal imaging, and quantification of on-resin recognition through UV, NMR, and CD experiments. (PDF)
