# Supplementary material for: On-Resin Recognition of Aromatic Oligopeptides and Proteins through Host-Enhanced Heterodimerization
Source: J Am Chem Soc. 2022 May 10;144(19):8474–9. doi: 10.1021/jacs.2c02287 (PMC9121384; doi:10.1021/jacs.2c02287)
Supplement: Supplementary file 1 — ja2c02287_si_001.pdf [file ja2c02287_si_001.pdf]

Supporting Information: On-Resin Recognition of Aromatic Oligopeptides and Proteins through Host-Enhanced Heterodimerization

Xiaoyi Chen[†,‡], Zehuan Huang[†,‡], Renata L. Sala[†], Alan M. McLean[†], Guanglu Wu[†], Kamil Sokołowski[†], Katherine King[†], Jade A. McCune[†], and Oren A. Scherman[†,*]

Melville Laboratory for Polymer Synthesis, Yusuf Hamied Department of Chemistry, University of Cambridge, Lensfield Road, Cambridge CB2 1EW, UK.

[‡]X.C. and Z.H. contributed equally to this work

E-mail: oas23@cam.ac.uk

Contents

1 Materials & instrumentation S2

2 Synthesis & characterization of model peptides S6

3 Study on heteropeptide dimerization by ^{1}H &^{19}F NMR S11

4 Thermodynamic investigations by ITC S22

5 Statistical analysis on confocal imaging S27

6 Quantification of on-resin recognition by UV, ^{1}H NMR & CD S29

References S38

1 Materials & instrumentation

Materials Unless noted, all the starting materials were purchased from commercial suppliers and were used without further purification: (L)-Fmoc-Glu(OtBu)-OH, (L)-Fmoc-Gly-OH, (L)-Fmoc-Leu-OH, (L)-Fmoc-Lys(Boc)-OH, (L)-Fmoc-Phe-OH, (L)-Fmoc-Trp(Boc)-OH, (L)-Fmoc-Tyr(tBu)-OH, (L)-Fmoc-3-(2-naphthyl)-alanine-OH, piperidine, diethyl ether, (L)-Fmoc-pentafluorophenylalanine-OH, diisopropylethylamine (DIPEA), trifluoroacetic acid (TFA), triisopropylsilane (TIPS), N,N,N',N'-tetramethyl-O-(1H-benzotriazol-1-yl)uronium hexafluorophosphate (HBTU), dimethylformamide (DMF), dichloromethane (DCM), monobasic sodium phosphate (NaH_2PO_4), dibasic sodium phosphate (Na_2HPO_4), deuterium oxide (D_2O, D, 99.8%), 1,3-dimethylaminoadamantane hydrochloride (memantine hydrochloride, DMADA), Rink Amide-AM Resin, H-Rink Amide ChemMatrix Resin (surface loading of functionalization $\approx$ 0.5 mmol/g), Insulin (Human Recombinant). Phosphate buffer solution was prepared by adding 10 mM NaH_2PO_4 solution to 10 mM Na_2HPO_4 solution until pH equal to 7.0. Cucurbit[8]uril (CB[8]) was synthesized and purified according to a previous report,[S1] and the molecular weight for CB[8] was calibrated as 1600 g/mol. Water was obtained from a Milli-Q Integral Water Purification System (22.5 MΩ·cm).

Peptide Synthesis and Purification All the tripeptides (XGG, GXG, GGX) were synthesized on an automated microwave peptide synthesizer (Liberty Blue, CEM) using standard 9-fluorenylmethoxycarbonyl (Fmoc) chemistry. Peptides were cleaved from the Rink Amide-AM resin with a mixture of 95% TFA, 2.5% TIPS, and 2.5% H_2O and shaken for 1 hr at room temperature. The resin was filtered and washed with the cleavage mixture. After evaporating most of the cleavage mixture, the peptides were crushed out and washed three times with cold diethyl ether. The crude peptides were purified using reverse-phase high-performance liquid chromatography (RP-HPLC) on a Phenomenex C18 Kinetic-Evo column with 5 μm pore size, 110 Å particle size and 150 × 21.2 mm dimensions. The mobile phase for water contained 0.1% TFA. The purification method gradient was set from 5% acetonitrile and 95% water to 100% acetonitrile. Dry peptides were obtained (as TFA salt) by lyophilization and then verified by NMR and ESI-MS. On resin peptide F'GGGGG were synthesized using H-Rink Amide ChemMatrix Resin using the same method above. The F'GGGGG-functionalized resin was washed with DMF for 3 times and then left in D_2O/H_2O (for 3 days, change with fresh D_2O/H_2O each day). Then, the washed F'GGGGG resin was preserved in D_2O/H_2O in the fridge at 277 K.

Nuclear Magnetic Resonance Spectroscopy (NMR) ^{1}H, ^{13}C & ^{19}F NMR spectra of pure peptides were acquired in D_2O at 298 K on a Bruker AVANCE 400 (400 MHz) apparatus and a Bruker AVANCE 500 apparatus with TCI Cryoprobe (500 MHz). ^{1}H & ^{19}F NMR spectra of other experiments (NMR titration, on-resin recognition tests) were acquired in phosphate buffer (D_2O) with pH 7.0 on a Bruker AVANCE 400 (400 MHz) apparatus. Chemical shifts were referenced to the residual solvent peak of HDO at 4.79 ppm.

Isothermal Titration Calorimetry (ITC) ITC experiments were operated on a Malvern MicroCal Auto-ITC200 apparatus at 298 K in 10 mM phosphate buffer with pH 7.0. In a

typical experiment for heteroternary complexation, the 1:1 mixture of CB[8] and F'GG was loaded in the sample cell with a concentration of 0.2 mM, and the second guest peptide was loaded in the syringe with a concentration of 3.0 mM. One titration experiment consisted of 1 injection of 0.6 μL and 32 consecutive injections of 1.2 μL with 90 s intervals between injections. The first data point was removed before analysis. The ITC data were fitted by MicroCal Analysis Centre software using one set of sites model. In a typical experiment for homoternary complexation, the host molecule (CB[8]) was loaded in the sample cell with a concentration of 0.1 mM, and the guest peptide was loaded in the syringe with a concentration of 2.0 mM. The injection and interval settings were the same as above. The first data point was removed from the data set before analysis. The obtained ITC curves were fitted by MicroCal Analysis Centre software using sequential binding model. All parameters were averaged with three replicates, s.d. n = 3. K_a values $< 10^2$ M^{-1} were too low to be detected.

Electrospray Ionization Mass Spectrometry (ESI-MS) ESI-MS data were obtained on a Thermo Fisher Q Exactive Orbitrap mass spectrometry with a nanospraying ion source. Positive mode was chosen for all experiments at a working temperature of 320 °C and a capillary voltage of 1.5 kV. All the sample solutions were prepared in pure water.

Ultraviolet (UV) Spectroscopy UV spectra were obtained on a Cary 4000 Ultraviolet-visible Spectrometer. A pair of quartz cuvettes with 2 mm gap was used. Double beam mode with a fixed 2 nm spectral bandwidth and baseline correction were selected. In a typical run, 300 μl of 1 mM solution was transferred to the quartz cuvette and was scanned compared to phosphate buffer in D_2O. UV spectra were obtained in the linear absorption range (0.2–0.8) for better quantification.

Confocal Microscopy On-resin imaging was performed on a Leica Stellaris 5 confocal microscope (Leica Microsystems) with a 40x oil immersion objective and numerical aperture of 1.3. Suspensions of resin beads were dropped on a coverslip and stacks of 50 images with a resolution of 1024 x1024 pixels at λ_{EXC} 405 nm were acquired. The stack boundaries were selected at the point where there was no distinguishable difference of signal-to-noise for both extremes (edges) of the beads. The 3D dataset was processed by Fiji/ImageJ and the maximum intensity Z-projection was obtained to compare the mean fluorescent intensity across resin beads. 3 groups of resin beads were tested: A group, original unmodified ChemMatrix resin (resin) and F'G_5 functionalized resin (resin-G_5F'); B group, resin-G_5F' with WGG but no CB[8] (resin-G_5F' + WGG), resin-G_5F' with WGG and CB[8] (resin-G_5F' + WGG + CB[8]), resin-G_5F' with WGG, CB[8] and then DMADA solution (resin-G_5F' + WGG + CB[8] + DMAMA); C group, resin-G_5F' with WG_5-dansyl but no CB[8] (resin-G_5F' + WG_5-dansyl), resin-G_5F' with WG_5-dansyl and CB[8] (resin-G_5F' + WG_5-dansyl + CB[8]), resin-G_5F' with WG_5-dansyl, CB[8] and then DMADA solution (resin-G_5F' + WG_5-dansyl + CB[8] + DMAMA). 3 groups of resin beads were prepared according to the procedures in the UV Spectroscopy above. All samples then were drained and added with the same amount of phosphate buffer with pH 7.0 to yield suspensions (10 mM) for imaging.

Circular Dichroism (CD) Spectroscopy CD measurements were performed on a Chirascan spectrometer (Applied Photophysics). Scans were carried out at 298 K from 200–500 nm at 1.0 nm intervals. The acquisition time was 0.5 second in a 0.1 cm path length quartz cuvette. CD spectra were collected using solutions from the above UV experiments of the 8-day storage and release experiments. The original insulin solution was 0.2 mM. All solutions were diluted 2x before the measurements. All scans were collected and averaged out over three scans with background noise correction.

Procedures for On-Resin Recognition A standard procedure of NMR quantification for on-resin recognition within a peptide mixture is shown as follows. A solution of mixed peptides containing WGG, KGG, LGG, EGG, CB[8] ([XGG] = CB[8] = 1 mM) was used as the original cycle 0 solution. For cycle 1, 1.0 ml of the mixed peptides (1 mM) was added to the F'GGGGG-functionalized resin (10 mM) and shaken for 10 min at room temperature. After draining, 1 ml of 2 mM DMADA solution was added, shaken for 45 min, and collected as the released solution. For cycle 2, fresh CB[8] (2.88 mg, 1.8 μmol) was added to 0.9 ml of the released solution (2 mM DMADA, 1.8 μmol) from cycle 1 to bind with excess DMADA and the isolated WGG. Around 0.1 ml solution was lost after each cycle. This new solution was added with the resin, shaken for 45 min, drained and then 0.9 ml of 2 mM DMADA solution was added to generate the released solution from cycle 2. For cycle 3, fresh CB[8] (2.56 mg, 1.6 μmol) was added to 0.8 ml of the released solution (2 mM DMADA, 1.6 μmol) from cycle 2. This new solution was added with the resin, shaken for 45 min, drained and then 0.8 ml of DMADA solution was added to generate the released solution from cycle 3. The original cycle 0 solution and the subsequently released solutions from each cycle were tested with NMR. After NMR analysis, the relative amount of WGG was determined by integrating a set of doublet peak at 3.36 ppm; the relative amount of KGG was determined by integrating a set of triplet peak at 3.02 ppm; the relative amount of EGG was determined by integrating a set of triplet peak at 2.37 ppm. There was a singlet peak of KGG, LGG, EGG that overlapped at 3.95 ppm. Thus, the relative amount of LGG was determined by the relative amount of KGG and EGG subtracted from the integration of peak at 3.95 ppm.

A standard procedure of UV quantification for peptide on-resin recognition is shown as follows. Using a model tripeptide WGG as an example, 10 μmol F'GGGGG equivalent of resin D_2O suspension was added to a 12 ml syringe with a polyester frit (r = 8.0 mm, h = 2.0 mm). After D_2O was drained, 1 ml of 1 mM WGG:CB[8] = 1:1 solution (original solution, in D_2O, phosphate buffer, pH = 7.0) was added to the resin. Then, the plunge and a stopper were assembled to the syringe to form a sealed chamber. The syringe was transferred onto a wrist-action shaker and shaken for 10 min. Then, the liquid (resin-treated solution) in syringe was drained thoroughly and the resin was washed with D_2O once. After that, 1 ml of 2 mM DMADA solution (in D_2O, phosphate buffer, pH = 7.0) was added to the resin and shaken for an additional 45 min. The solution (released solution) was drained for further testing. For efficiency test over 3 cycles (Figure 4c), WGG:CB[8] = 1:1 solution and DMADA solution were alternatively added to resin, shaken and drained for three cycles and produced a series of resin-treated and released solutions.

A standard procedure of UV quantification for insulin on-resin recognition is shown as follows. Insulin (Human Recombinant) was suspended at 0.2 mM using 0.22 μm filtered

H_2O. 1 N HCl was immediately added to reach acid concentration of 5 mM. Solution was gently stirred until fully dissolved and was filtered through 0.22 μm filter and stored at 277 K. Method was slightly modified from a Cold Spring Harbor Protocol.[S2] Insulin solution was warmed to room temperature before being used in on-resin recognition. In a typical experiment, 8 μmol F'GGGGG equivalent of resin H_2O suspension was added to a 12 ml syringe with a polyester frit (r = 8.0 mm, h = 2.0 mm). After H_2O was drained, 8 ml of 0.1 mM CB[8] solution was added to the resin and the plunge and a stopper were assembled to the syringe to form a sealed chamber. The syringe was transferred onto a wrist-action shaker and shaken for 12 hours. Then, after CB[8] solution was drained and resin was washed once with H_2O, 0.8 ml of 0.2 mM insulin solution was added to the resin and shaken gently for 3 hours. Then, the liquid (resin-treated solution) in syringe was drained thoroughly and the resin was washed with H_2O once. After that, 0.8 ml of 2 mM DMADA solution (in 5 mM of HCl) was added to the resin and shaken gently for an additional 3 hours. The solution (released solution) was drained. The origin insulin solution, resin-treated solution and released solution were measured on UV spectroscopy immediately.

The 8-day storage and release experiment was conducted as follows. 9 syringes were prepared each with 8 μmol F'GGGGG equivalent of resin H_2O suspension. On day 0, as the standard procedure of UV quantification for insulin on-resin recognition described above, all 9 syringes were drained, bound with CB[8], washed, bound with insulin, drained (to get resin-treated solutions), washed. All syringes with the resin were kept at room temperature. Starting from day 0 to day 8, on each day, a syringe with resin was added with 0.8 ml of 2 mM DMADA solution (to get released solution, one per day). All yielded solutions were analyzed on UV spectroscopy immediately. Numbers to calculate released percentage in Figure 4g were absorbance at 276 nm.

2 Synthesis & characterization of model peptides

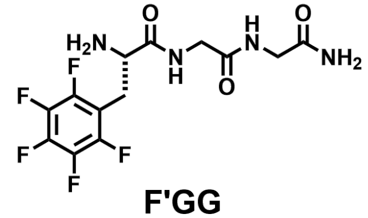

F'GG

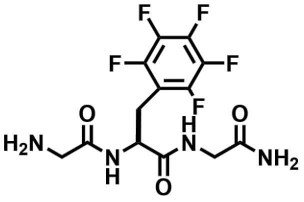

GF'G

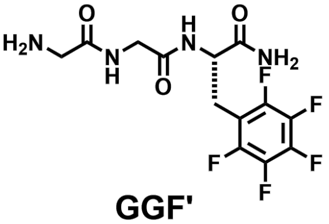

GGF'

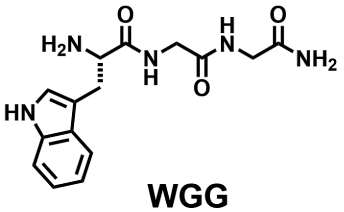

WGG

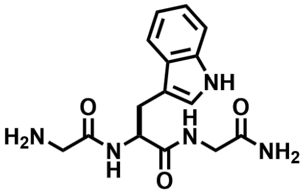

GWG

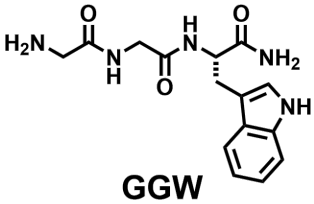

GGW

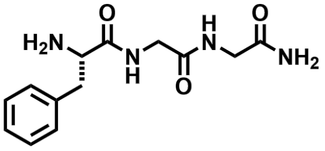

FGG

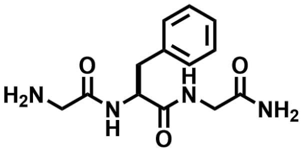

GFG

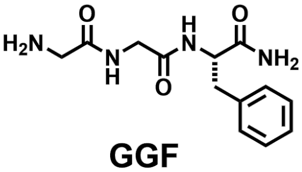

GGF

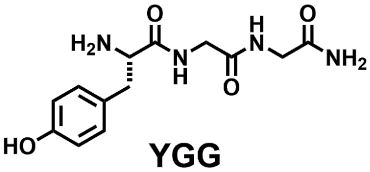

YGG

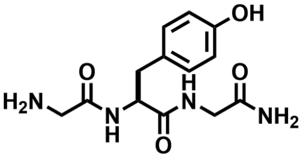

GYG

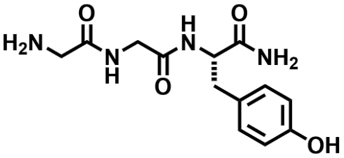

GGY

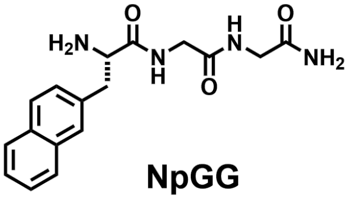

NpGG

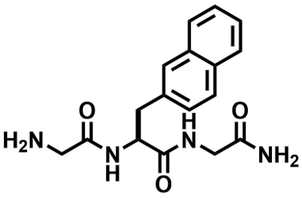

GNpG

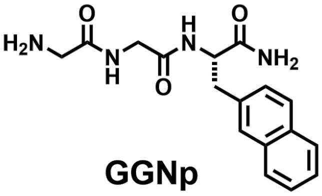

GGNp

(continued on next page)

(continued)

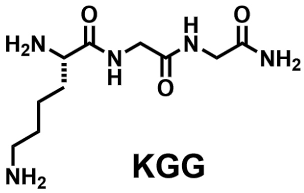

KGG

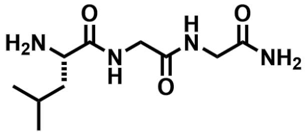

LGG

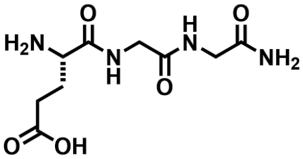

EGG

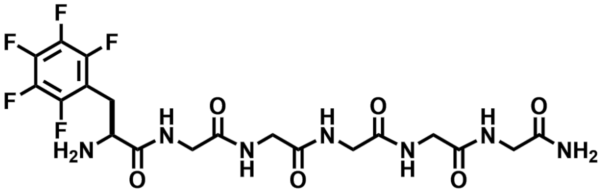

F'GGGGG

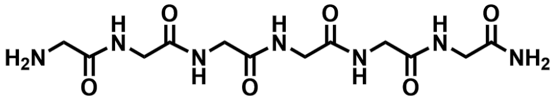

GGGGGG

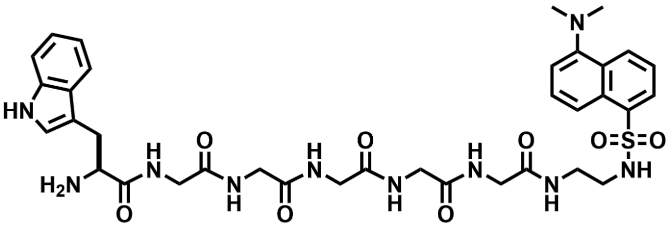

WGGGGG-dansyl

Chart S1: Molecular structures of all peptides used in this study. All these peptides are in TFA salt forms.

(S)-2-amino-N-(2-((2-amino-2-oxoethyl)amino)-2-oxoethyl)-3-(perfluorophenyl) propenamide (F'GG) ^{1}H NMR (500 MHz, D_2O, ppm): δ = 4.32 (t, 1H), 3.99 (q, 2H), 3.93 (s, 2H), 3.37 (d, 2H); ^{13}C NMR (125 MHz, D_2O): δ = 174.01, 170.89, 168.81, 146.39, 144.44, 141.90, 139.89, 138.49, 136.49, 107.29, 52.04, 42.30, 41.89, 23.85; ^{19}F NMR (375 MHz, D_2O, ppm): δ = -142.65, -154.41, -162.18; ESI MS for [M-H]+: calc. m/z = 369.10, found m/z = 369.17.

(S)-N-(2-amino-2-oxoethyl)-2-(2-aminoacetamido)-3-(perfluorophenyl) propanamide (GF'G) ^{1}H NMR (500 MHz, D_2O, ppm): δ = 4.77 – 4.74 (m, 1H), 3.95 – 3.86 (m, 2H), 3.85 – 3.79 (m, 2H), 3.32 (dd, 1H), 3.20 (dd, 1H); ^{13}C NMR (125 MHz, D_2O, ppm): δ = 173.56, 172.10, 166.91, 146.25, 144.32, 141.27, 139.28, 138.29, 136.33, 109.48, 52.55, 42.00, 40.22, 24.43; ^{19}F NMR (375 MHz, D_2O, ppm): δ = -143.48, -155.99, -162.89; ESI MS for [M-H]+: calc. m/z = 369.10, found m/z = 369.17.

(S)-2-(2-(2-aminoacetamido)acetamido)-3-(perfluorophenyl)propanamide (GGF') ^{1}H NMR (500 MHz, D_2O, ppm): δ = 4.73 – 4.70 (m, 1H), 3.96 (s, 2H), 3.88 (s, 2H), 3.31

(dd, 1H), 3.16 (dd, 1H); ^{13}C NMR (125 MHz, D_2O, ppm): δ = 174.32, 170.86, 167.64, 146.27, 144.34, 141.17, 139.17, 138.24, 136.27, 109.89, 52.04, 42.05, 40.28, 24.52; ^{19}F NMR (375 MHz, D_2O, ppm): δ = -143.47, -156.31, -163.04; ESI MS for [M-H]+: calc. m/z = 369.10, found m/z = 369.17.

(S)-2-amino-N-(2-((2-amino-2-oxoethyl)amino)-2-oxoethyl)-3-(1H-indol-3-yl) propanamide (WGG) ^{1}H NMR (500 MHz, D_2O, ppm): δ = 7.64 (d, 1H), 7.54 (d, 1H), 7.35 (s, 1H), 7.29 (t, 1H), 7.21 (t, 1H), 4.36 (t, 1H), 3.92 (d, 1H), 3.91 – 3.75 (m, 3H), 3.49 – 3.38 (m, 2H); ^{13}C NMR (125 MHz, D_2O, ppm): δ = 173.90, 171.22, 170.24, 136.11, 126.42, 125.23, 122.21, 119.61, 117.98, 112.03, 106.24, 53.68, 42.36, 41.83, 26.70; ESI MS for [M-H]+: calc. m/z = 318.16, found m/z = 318.34.

(S)-N-(2-amino-2-oxoethyl)-2-(2-aminoacetamido)-3-(1H-indol-3-yl)propanamide (GWG) ^{1}H NMR (500 MHz, D_2O, ppm): δ = 7.67 (d, 1H), 7.52 (d, 1H), 7.30 – 7.24 (m, 2H), 7.19 (t, 1H), 4.77 – 4.69 (m, 1H), 3.84 (d, 1H), 3.81 – 3.73 (m, 2H), 3.70 (d, 1H), 3.36 – 3.23 (m, 2H); ^{13}C NMR (125 MHz, D_2O, ppm): δ = 173.90, 173.77, 166.95, 136.04, 126.64, 124.54, 122.00, 119.38, 118.25, 111.89, 108.60, 54.89, 42.00, 40.23, 26.96; ESI MS for [M-H]+: calc. m/z = 318.16, found m/z = 318.34.

(S)-2-(2-(2-aminoacetamido)acetamido)-3-(1H-indol-2-yl)propanamide (GGW) ^{1}H NMR (500 MHz, D_2O, ppm): δ = 7.70 (d, 1H), 7.52 (d, 1H), 7.30 – 7.24 (m, 2H), 7.19 (t, 1H), 4.68 (dd, 1H), 3.99 – 3.87 (m, 2H), 3.77 (s, 2H), 3.34 (dd, 1H), 3.23 (dd, 1H); ^{13}C NMR (125 MHz, D_2O, ppm): δ = 176.11, 170.80, 167.56, 136.05, 126.84, 124.49, 121.89, 119.30, 118.29, 111.84, 108.86, 54.13, 42.11, 40.23, 26.96; ESI MS for [M-H]+: calc. m/z = 318.16, found m/z = 318.34.

(S)-2-amino-N-(2-((2-amino-2-oxoethyl)amino)-2-oxoethyl)-3-phenylpropanamide (FGG) ^{1}H NMR (500 MHz, D_2O, ppm): δ = 7.47 – 7.36 (m, 3H), 7.35 – 7.29 (m, 2H), 4.32 (t 1H), 4.01 (d, 1H), 3.94 – 3.87 (m, 3H), 3.29 – 3.20 (m, 2H); ^{13}C NMR (125 MHz, D_2O, ppm): δ = 173.97, 171.27, 169.82, 133.68, 129.35, 129.14, 128.00, 54.41, 42.26, 41.90, 36.69; ESI MS for [M-H]+: calc. m/z = 279.15, found m/z = 279.34.

(S)-N-(2-amino-2-oxoethyl)-2-(2-aminoacetamido)-3-phenylpropanamide (GFG) ^{1}H NMR (500 MHz, D_2O, ppm): δ = 7.44 – 7.37 (m, 2H), 7.37 – 7.31 (m, 1H), 7.34 – 7.28 (m, 2H), 4.68 (t, 1H), 3.86 (t, 2H), 3.81 – 3.74(m, 2H), 3.16 (dd, 1H), 3.07 (dd, 1H); ^{13}C NMR (125 MHz, D_2O, ppm): δ = 173.76, 173.49, 166.96, 136.05, 129.14, 128.80, 127.28, 55.32, 41.99, 40.18, 36.93; ESI MS for [M-H]+: calc. m/z = 279.15, found m/z = 279.34.

(S)-2-(2-(2-aminoacetamido)acetamido)-3-phenylpropanamide (GGF) ^{1}H NMR (500 MHz, D_2O, ppm): δ = 7.43 – 7.36 (m, 2H), 7.36 – 7.29 (m, 2H), 7.30 (t, 1H), 4.62 (dd, 1H), 3.94 (q, 2H), 3.85 (s, 2H), 3.19 (dd, 1H), 3.01 (dd, 1H); ^{13}C NMR (125 MHz, D_2O, ppm): δ = 175.70, 170.82, 167.57, 136.41, 129.14, 128.69, 127.11, 54.68, 42.01, 40.29, 36.98; ESI MS for [M-H]+: calc. m/z = 279.15, found m/z = 279.34.

(S)-2-amino-N-(2-((2-amino-2-oxoethyl)amino)-2-oxoethyl)-3-(4-hydroxyphenyl) propanamide (YGG) ^{1}H NMR (500 MHz, D_2O, ppm): δ = 7.22 – 7.15 (m, 2H), 6.93 – 6.87 (m, 2H), 4.26 (t, 1H), 4.05 – 3.97 (m, 1H), 3.96 – 3.88 (m, 3H), 3.16 (d, 2H); ^{13}C NMR (125 MHz, D_2O, ppm): δ = 173.94, 171.28, 169.89, 155.13, 130.78, 125.41, 115.81, 54.49, 42.26, 41.89, 35.86; ESI MS for [M-H]+: calc. m/z = 295.14, found m/z = 295.34.

(S)-N-(2-amino-2-oxoethyl)-2-(2-aminoacetamido)-3-(4-hydroxyphenyl) propanamide (GYG) ^{1}H NMR (500 MHz, D_2O, ppm): δ = 7.20 – 7.14 (m, 2H), 6.87 (m, 2H), 4.61 (t, 1H), 3.90 – 3.83 (m, 1H), 3.87 – 3.71 (m, 3H), 3.07 (dd, 1H), 3.00 (dd, 1H); ^{13}C NMR (125 MHz, D_2O, ppm): δ = 173.79, 173.59, 166.94, 154.52, 130.50, 127.82, 115.48, 55.51, 41.98, 40.19, 36.12; ESI MS for [M-H]+: calc. m/z = 295.14, found m/z = 295.25.

(S)-2-(2-(2-aminoacetamido)acetamido)-3-(4-hydroxyphenyl)propanamide (GGY) ^{1}H NMR (500 MHz, D_2O, ppm): δ = 7.20 – 7.13 (m, 2H), 6.90 – 6.83 (m, 2H), 4.56 (dd, 1H), 3.95 (q, 2H), 3.86 (s, 2H), 3.10 (dd, 1H), 2.94 (dd, 1H); ^{13}C NMR (125 MHz, D_2O, ppm): δ = 175.77, 170.79, 167.57, 154.37, 130.49, 128.22, 115.38, 54.84, 42.01, 40.30, 36.18; ESI MS for [M-H]+: calc. m/z = 295.14, found m/z = 295.34.

(S)-2-amino-N-(2-((2-amino-2-oxoethyl)amino)-2-oxoethyl)-3-(naphthalen-2-yl) propanamide (NpGG) ^{1}H NMR (500 MHz, D_2O, ppm): δ = 7.97 (t, 2H), 7.95 – 7.90 (m, 1H), 7.83 – 7.79 (m, 1H), 7.64 – 7.55 (m, 2H), 7.46 (dd, 1H), 4.44 – 4.39 (m, 1H), 3.89 (s, 2H), 3.74 – 3.62 (m, 2H), 3.44 (dd, 1H), 3.37 (dd, 1H); ^{13}C NMR (125 MHz, D_2O, ppm): δ = 173.74, 171.04, 169.72, 133.04, 132.38, 131.40, 128.84, 128.33, 127.72, 127.62, 126.92, 126.84, 126.61, 54.27, 42.23, 41.68, 36.87; ESI MS for [M-H]+: calc. m/z = 329.16, found m/z = 329.34.

(S)-N-(2-amino-2-oxoethyl)-2-(2-aminoacetamido)-3-(naphthalen-2-yl)propanamide (GNpG) ^{1}H NMR (500 MHz, D_2O, ppm): δ = 7.93 (td, 3H), 7.80 – 7.76 (m, 1H), 7.57 (tt, 2H), 7.46 (dd, 1H), 4.77 – 4.74(m, 1H), 3.84 (dd, 2H), 3.73 (dd, 2H), 3.32 (dd, 1H), 3.23 (dd, 1H); ^{13}C NMR (125 MHz, D_2O, ppm): δ = 173.67, 173.45, 166.94, 133.76, 133.04, 132.10, 128.31, 127.75, 127.62, 127.47, 127.24, 126.62, 126.21, 55.25, 41.96, 40.16, 37.13; ESI MS for [M-H]+: calc. m/z = 329.16, found m/z = 329.34.

(S)-2-(2-(2-aminoacetamido)acetamido)-3-(naphthalen-2-yl)propanamide (GGNp) ^{1}H NMR (500 MHz, D_2O, ppm): δ = 7.97 – 7.89 (m, 3H), 7.79 – 7.75 (m, 1H), 7.57 (tt, 2H), 7.46 (dd, 1H), 4.73 (dd, 1H), 3.90 (q, 2H), 3.76 (s, 2H), 3.36 (dd, 1H), 3.16 (dd, 1H); ^{13}C NMR (125 MHz, D_2O, ppm): δ = 175.66, 170.78, 167.47, 134.17, 133.03, 132.06, 128.19, 127.71, 127.61, 127.49, 127.31, 126.51, 126.11, 54.53, 42.02, 40.21, 37.16; ESI MS for [M-H]+: calc. m/z = 329.16, found m/z = 329.34.

(S)-2,6-diamino-N-(2-((2-amino-2-oxoethyl)amino)-2-oxoethyl)hexanamide (KGG) ^{1}H NMR (500 MHz, D_2O, ppm): δ = 4.12 – 4.02 (m, 3H), 3.95 (s, 2H), 3.02 (t, 2H), 2.01 – 1.90 (m, 2H), 1.73 (p, 2H), 1.55 – 1.43 (m, 2H); ^{13}C NMR (125 MHz, D_2O, ppm): δ =

174.05, 171.36, 170.30, 52.94, 42.19, 41.93, 38.93, 30.18, 26.29, 21.06; ESI MS for [M-H]+: calc. m/z = 260.17, found m/z = 260.34.

(S)-2-amino-N-(2-((2-amino-2-oxoethyl)amino)-2-oxoethyl)-4-methylpentanamide (LGG) ^{1}H NMR (500 MHz, D_2O, ppm): δ = 4.13 – 4.06 (m, 2H), 4.04 – 3.99 (m, 1H), 3.95 (s, 2H), 1.84 – 1.66 (m, 3H), 0.98 (t, 6H); ^{13}C NMR (125 MHz, D_2O, ppm): δ = 174.05, 171.42, 171.16, 51.85, 42.28, 41.92, 39.71, 23.78, 21.59, 20.96; ESI MS for [M-H]+: calc. m/z = 245.16, found m/z = 245.34.

(S)-4-amino-5-((2-((2-amino-2-oxoethyl)amino)-2-oxoethyl)amino)-5-oxopentanoic acid (EGG) ^{1}H NMR (500 MHz, D_2O, ppm): δ = 4.15 (t, 1H), 4.09 (d, 1H), 4.02 (d, 1H), 3.95 (s, 2H), 2.60 (t, 2H), 2.21 (q, 2H); ^{13}C NMR (125 MHz, D_2O, ppm): δ = 176.18, 174.06, 171.35, 169.95, 52.40, 42.28, 41.93, 29.05, 25.74; ESI MS for [M-H]+: calc. m/z = 261.12, found m/z = 261.34.

(S)-2-amino-N-(14-amino-2,5,8,11,14-pentaoxo-3,6,9,12-tetraazatetradecyl)-3-(perfluorophenyl) propenamide (F'GGGGG) ^{1}H NMR (700 MHz, D_2O, ppm): δ = 4.30 (t, 1H), 4.02 (m, 8H), 3.95 (s, 2H), 3.37 (d, 2H); ^{19}F NMR (375 MHz, D_2O, ppm): δ = -142.63, -154.54, -162.22; ESI MS for [M-H]+: calc. m/z = 540.16, found m/z = 540.67.

2-amino-N-(14-amino-2,5,8,11,14-pentaoxo-3,6,9,12-tetraazatetradecyl)acetamide (GGGGGG) ^{1}H NMR (700 MHz, D_2O, ppm): δ = 4.09 (s, 2H), 4.04 (d, 4H), 4.02 (s, 2H), 3.95 (s, 2H), 3.93 (s, 2H); ESI MS for [M-H]+: calc. m/z = 360.16, found m/z = 360.50.

(S)-2-amino-N-(17-((5-(dimethylamino)naphthalene)-1-sulfonamido)-2,5,8,11,14-pentaoxo-3,6,9,12,15-pentaazaheptadecyl)-3-(1H-indol-3-yl)propenamide (WGGGGG-dansyl) ^{1}H NMR (700 MHz, D_2O, ppm): δ = 8.68 (d, 1H), 8.47 (d, 1H), 8.36 (d, 1H), 7.97 – 7.85 (m, 3H), 7.62 (d, 1H), 7.53 (d, 1H), 7.35 (s, 1H), 7.26 (t, 1H), 7.17 (t, 1H), 4.41 (t, 1H), 4.07 (s, 2H), 4.03 (s, 2H), 4.02 – 3.92 (m, 4H), 3.69 (s, 2H), 3.48 – 3.40 (m, 2H), 3.37 (s, 6H), 3.21 (t, 2H), 3.14 (t, 2H); ESI MS for [M-H]+: calc. m/z = 765.31, found m/z = 765.34.

3 Study on heteropeptide dimerization by ^{1}H &^{19}F NMR

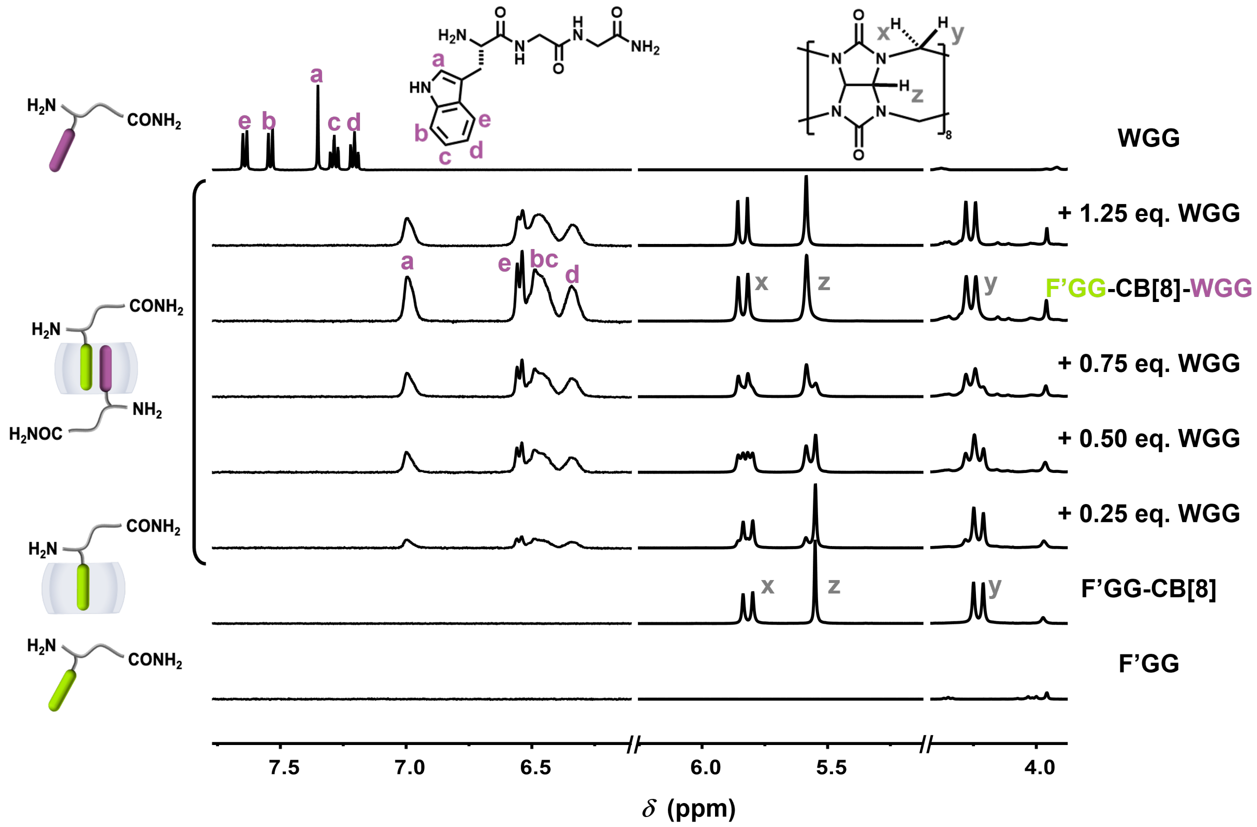

Figure S1: ^{1}H NMR spectra obtained through titrations of WGG into F'GG-CB[8] (1:1 complex) at the molar ratio ranging from 0.25:1.00 to 1.25:1.00 with three controls of free F'GG, pure F'GG-CB[8] complex, free WGG. Within each area (7.6 - 6.4 ppm, 5.8 – 5.6 ppm, 4.4 – 3.8 ppm), the intensity of free F'GG was adjusted by a factor of 1000X and free WGG by 870x. Among these areas, the intensity of all spectra in 7.6 – 6.4 ppm area was adjusted by a factor of 27x (400 MHz, 298 K, D_2O, phosphate buffer, pH = 7.0)

As shown in Figure S1, by adding WGG to F'GG-CB[8] (1:1 complex), the aromatic proton peaks from 7.6 ppm to 7.2 ppm shifted to 7.0 ppm to 6.3 ppm and became blunt because of the encapsulation. The proton x, y, z on CB[8] also shifted from one state to another. This observation indicated that a new heteroternary complex of WGG-F'GG-CB[8] was formed.

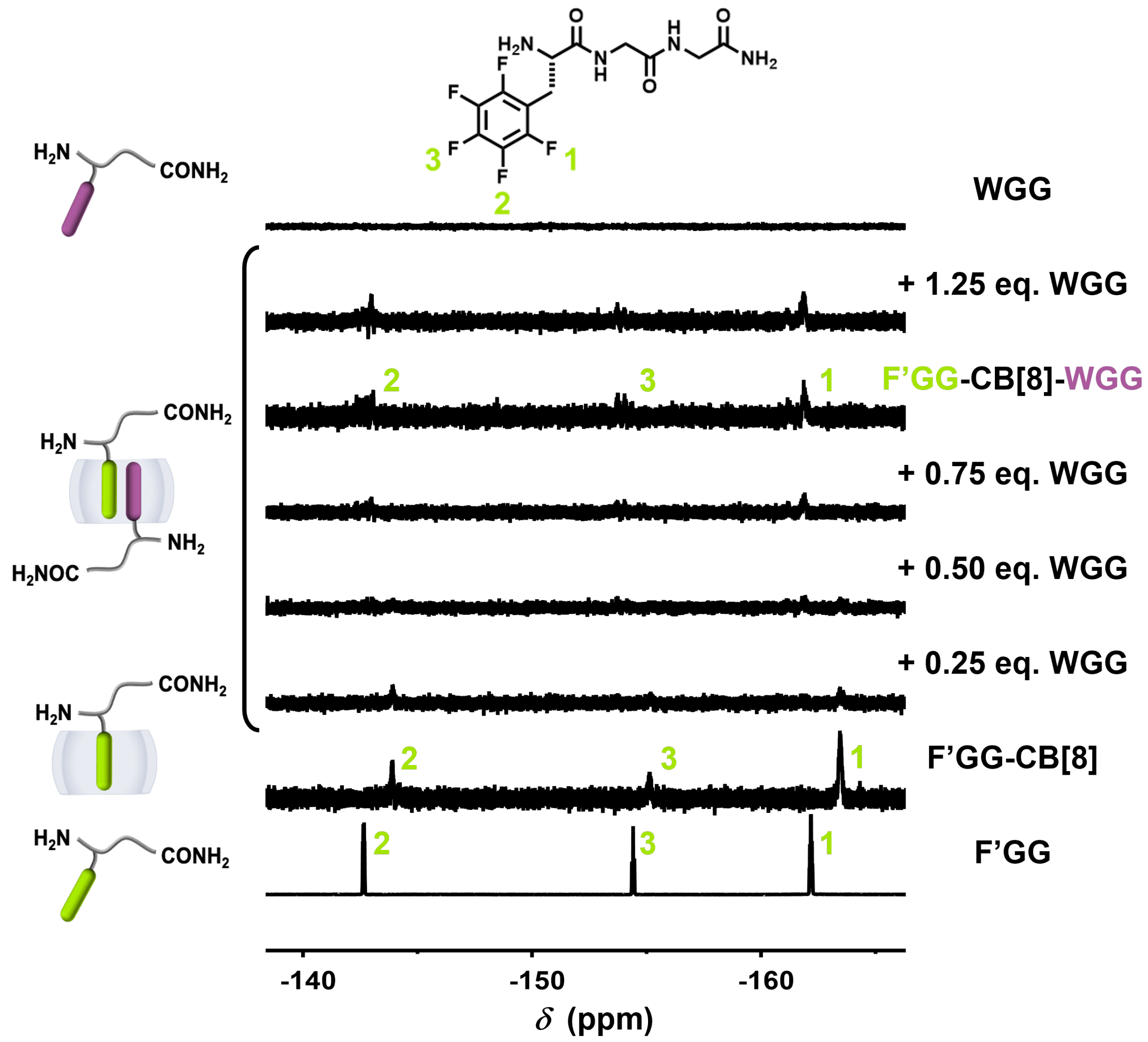

Figure S2: ^{19}F NMR spectra obtained through titrations of WGG to F'GG-CB[8] (1:1 complex) at the molar ratio ranging from 0.25:1.00 to 1.25:1.00 with three controls of free F'GG, pure F'GG-CB[8] complex, free WGG. The intensity of free F'GG was adjusted by a factor 0.01 x (400 MHz, 298 K, D_2O, phosphate buffer, pH = 7.0)

As shown in Figure S2, by adding WGG to F'GG-CB[8] (1:1 complex), the peaks of fluorine atoms 1, 2, 3 at -162.2, -142.6, -154.4 ppm shifted downfield and then gradually moved upfield along with the addition of WGG. This observation is consistent with Figure S1 and indicated a new heteroternary complex of WGG-F'GG-CB[8] was formed.

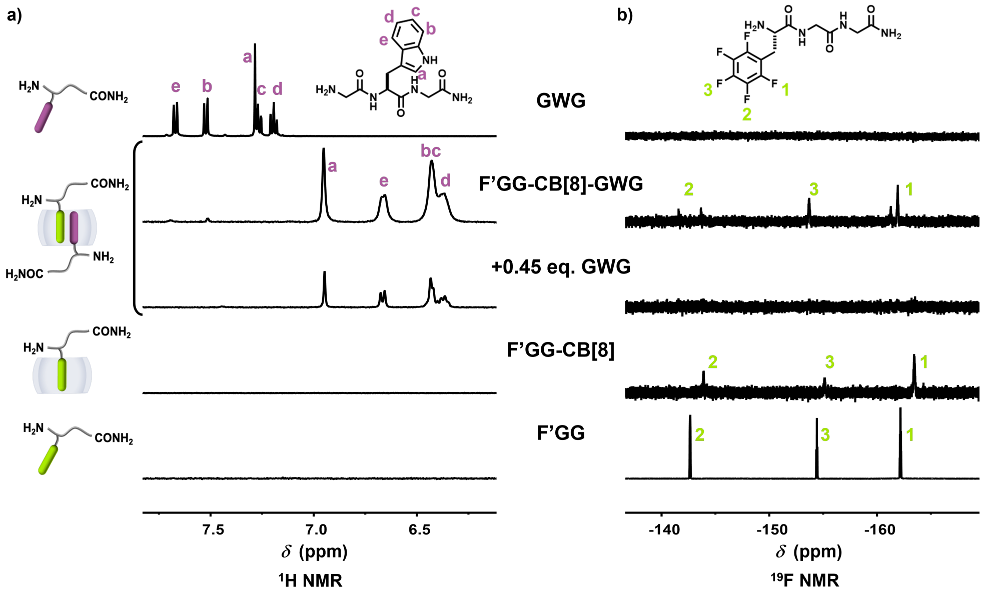

Figure S3: a) ^{1}H and b) ^{19}F NMR spectra obtained through titrations of GWG to F'GG-CB[8] (1:1 complex) at the molar ratio 0.45:1.00 and 1.00:1.00 with three controls of free F'GG, pure F'GG-CB[8] complex and free GWG. The ^{1}H NMR spectrum intensity of free GWG was adjusted by a factor of 100 x and ^{19}F NMR spectrum intensity of free F'GG by 0.0002 x. (400 MHz, 298 K, D_2O, phosphate buffer, pH = 7.0)

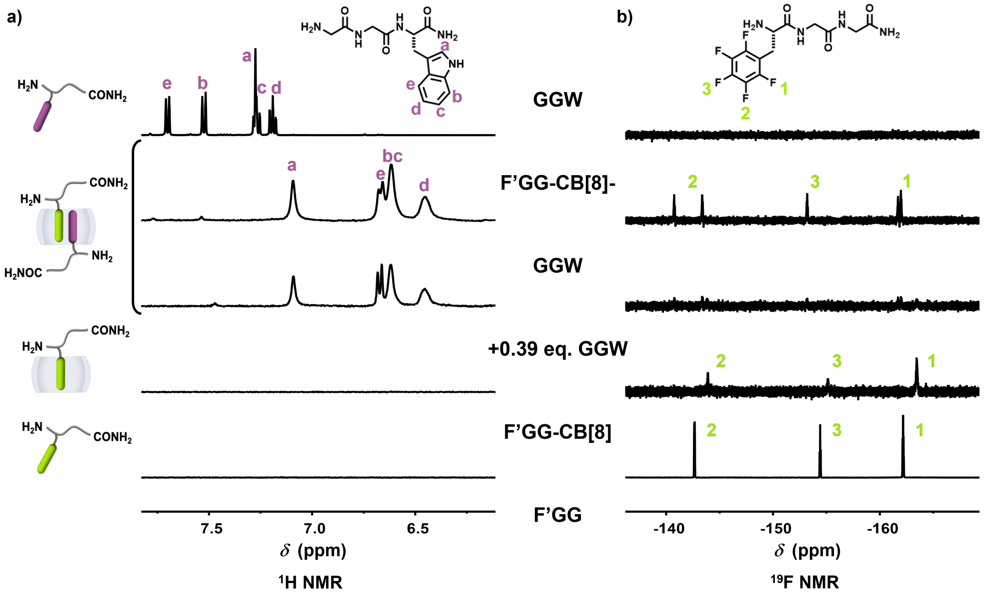

Figure S4: a) ^{1}H and b) ^{19}F NMR spectra obtained through titrations of GGW to F'GG-CB[8] (1:1 complex) at the molar ratio 0.39:1.00 and 1.00:1.00 with three controls of free F'GG, pure F'GG-CB[8] complex and free GGW. The ^{1}H NMR spectrum intensity of free GGW was adjusted by a factor of 73 x and ^{19}F NMR spectrum intensity of free F'GG by 0.0002 x. (400 MHz, 298 K, D_2O, phosphate buffer, pH = 7.0)

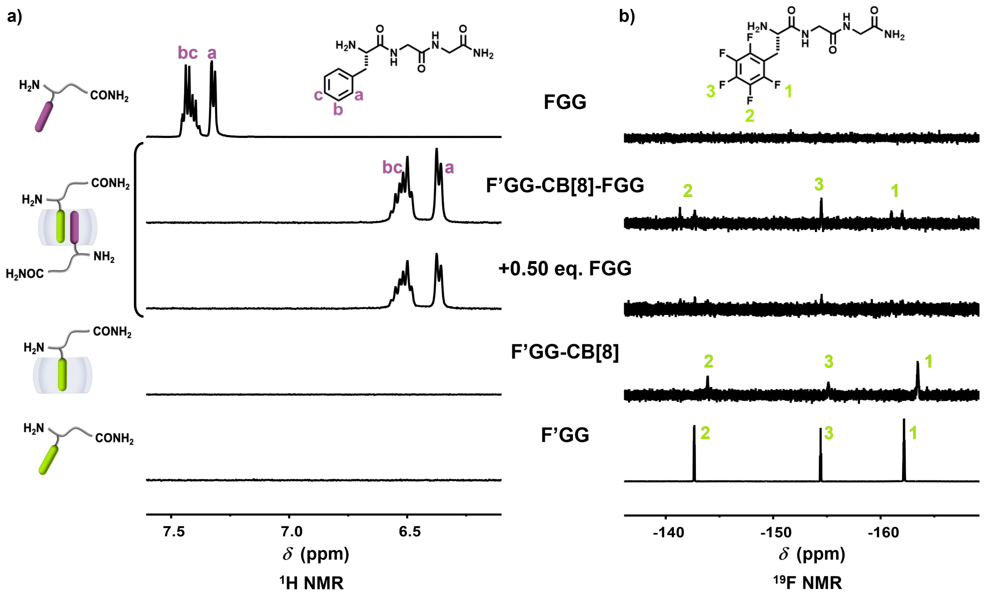

Figure S5: a) ^{1}H and b) ^{19}F NMR spectra obtained through titrations of FGG to F'GG-CB[8] (1:1 complex) at the molar ratio 0.50:1.00 and 1.00:1.00 with three controls of free F'GG, pure F'GG-CB[8] complex and free FGG. The ^{1}H NMR spectrum intensity of free FGG was adjusted by a factor of 80 x and ^{19}F NMR spectrum intensity of free F'GG by 0.0002 x. (400 MHz, 298 K, D_2O, phosphate buffer, pH = 7.0)

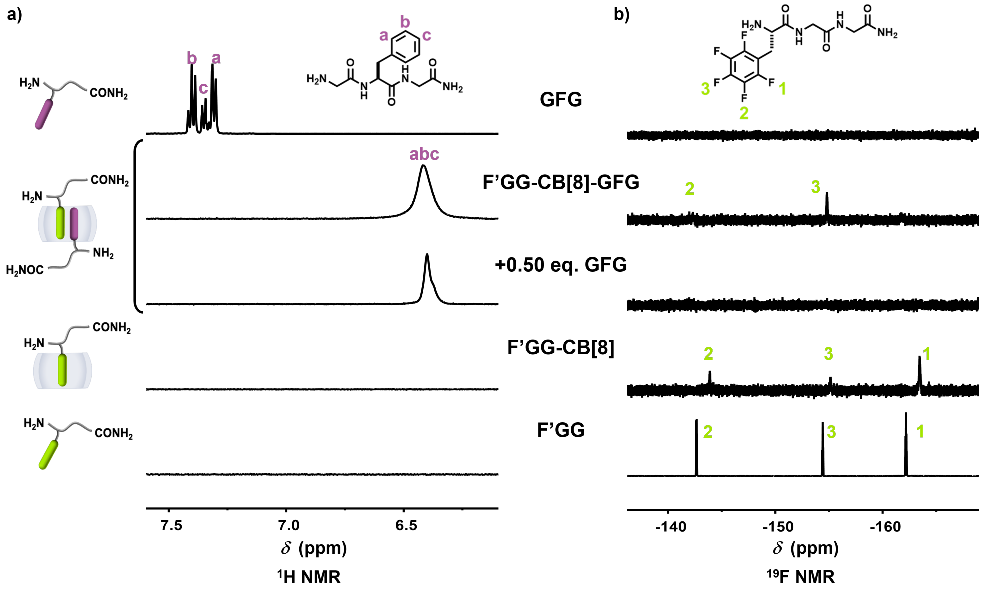

Figure S6: a) ^{1}H and b) ^{19}F NMR spectra obtained through titrations of GFG to F'GG-CB[8] (1:1 complex) at the molar ratio 0.50:1.00 and 1.00:1.00 with three controls of free F'GG, pure F'GG-CB[8] complex and free GFG. The ^{1}H NMR spectrum intensity of free GFG was adjusted by a factor of 22 x and ^{19}F NMR spectrum intensity of free F'GG by 0.0002 x. (400 MHz, 298 K, D_2O, phosphate buffer, pH = 7.0)

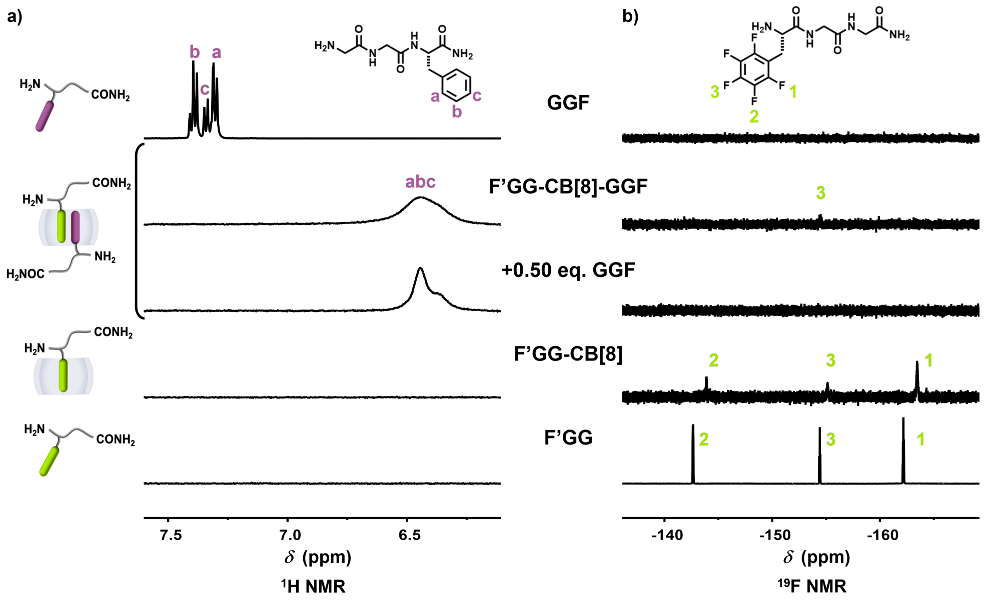

Figure S7: a) ^{1}H and b) ^{19}F NMR spectra obtained through titrations of GGF to F'GG-CB[8] (1:1 complex) at the molar ratio 0.50:1.00 and 1.00:1.00 with three controls of free F'GG, pure F'GG-CB[8] complex and free GGF. The ^{1}H NMR spectrum intensity of free GGF was adjusted by a factor of 9 x and ^{19}F NMR spectrum intensity of free F'GG by 0.0002 x. (400 MHz, 298 K, D_2O, phosphate buffer, pH = 7.0)

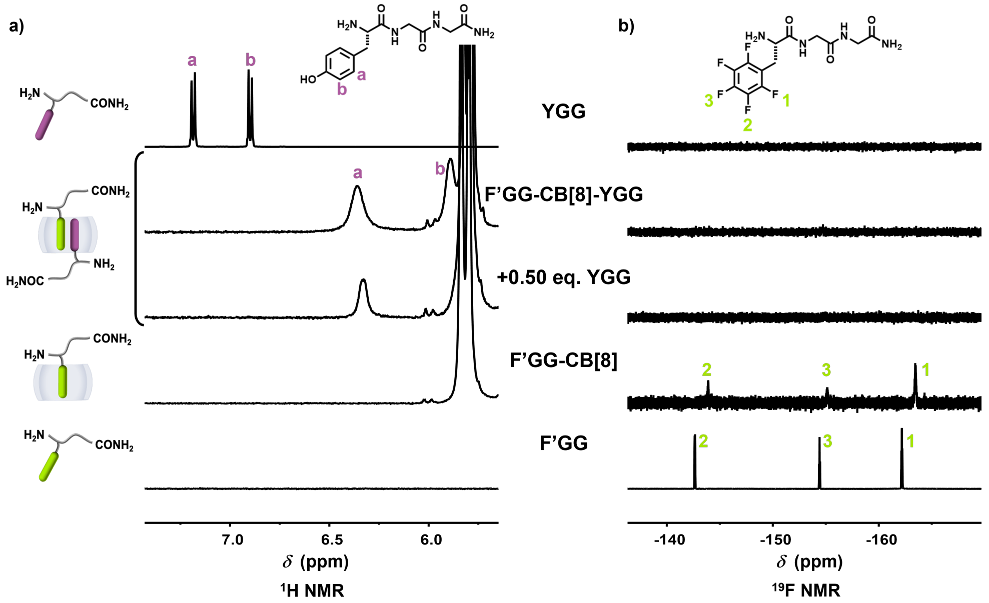

Figure S8: a) ^{1}H and b) ^{19}F NMR spectra obtained through titrations of YGG to F'GG-CB[8] (1:1 complex) at the molar ratio 0.50:1.00 and 1.00:1.00 with three controls of free F'GG, pure F'GG-CB[8] complex and free YGG. The ^{1}H NMR spectrum intensity of free YGG was adjusted by a factor of 20 x and ^{19}F NMR spectrum intensity of free F'GG by 0.0002 x. (400 MHz, 298 K, D_2O, phosphate buffer, pH = 7.0)

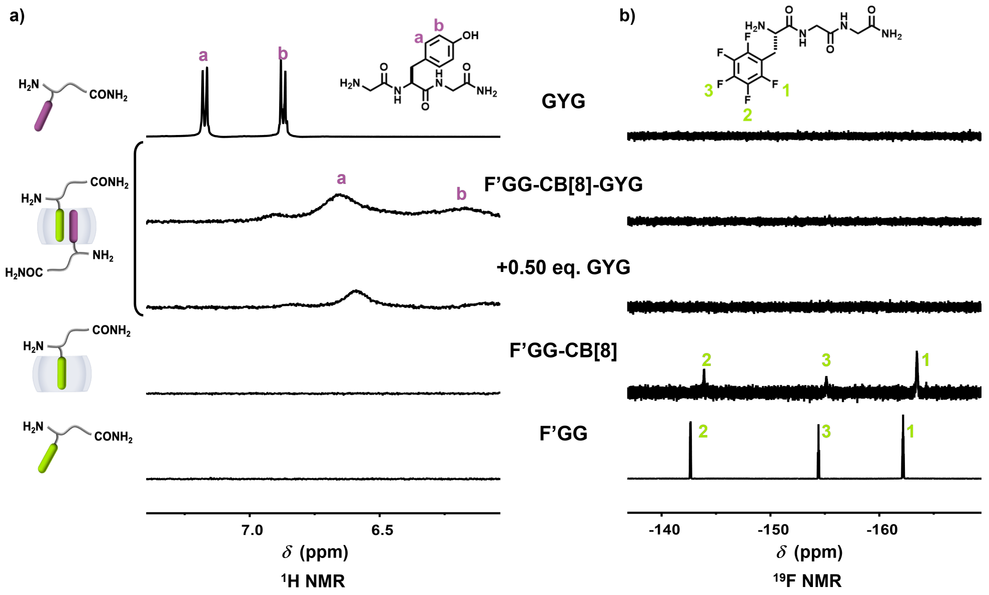

Figure S9: a) ^{1}H and b) ^{19}F NMR spectra obtained through titrations of GYG to F'GG-CB[8] (1:1 complex) at the molar ratio 0.50:1.00 and 1.00:1.00 with three controls of free F'GG, pure F'GG-CB[8] complex and free GYG. The ^{1}H NMR spectrum intensity of free GYG was adjusted by a factor of 6 x and ^{19}F NMR spectrum intensity of free F'GG by 0.0002 x. (400 MHz, 298 K, D_2O, phosphate buffer, pH = 7.0)

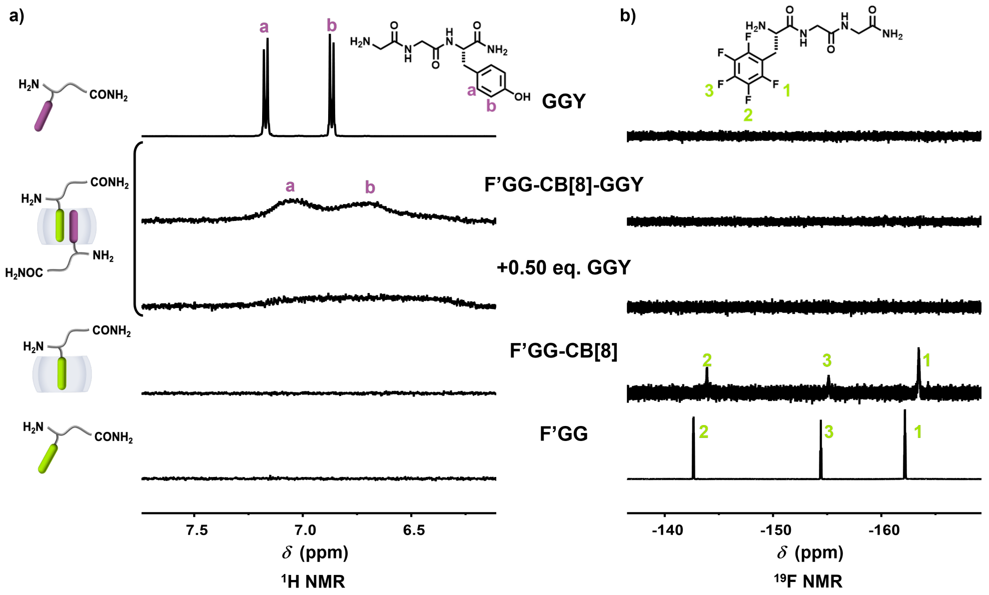

Figure S10: a) ^{1}H and b) ^{19}F NMR spectra obtained through titrations of GGY to F'GG-CB[8] (1:1 complex) at the molar ratio 0.50:1.00 and 1.00:1.00 with three controls of free F'GG, pure F'GG-CB[8] complex and free GGY. The ^{1}H NMR spectrum intensity of free GGY was adjusted by a factor of 6 x and ^{19}F NMR spectrum intensity of free F'GG by 0.0002 x. (400 MHz, 298 K, D_2O, phosphate buffer, pH = 7.0)

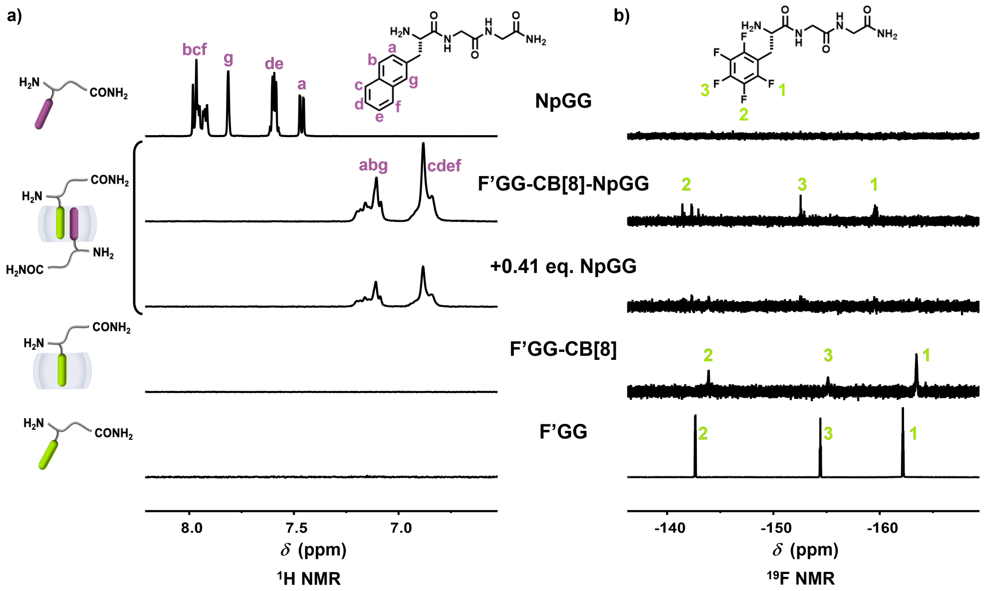

Figure S11: a) ^{1}H and b) ^{19}F NMR spectra obtained through titrations of NpGG to F'GG-CB[8] (1:1 complex) at the molar ratio 0.41:1.00 and 1.00:1.00 with three controls of free F'GG, pure F'GG-CB[8] complex and free NpGG. The ^{1}H NMR spectrum intensity of free NpGG was adjusted by a factor of 61 x and ^{19}F NMR spectrum intensity of free F'GG by 0.0002 x. (400 MHz, 298 K, D_2O, phosphate buffer, pH = 7.0)

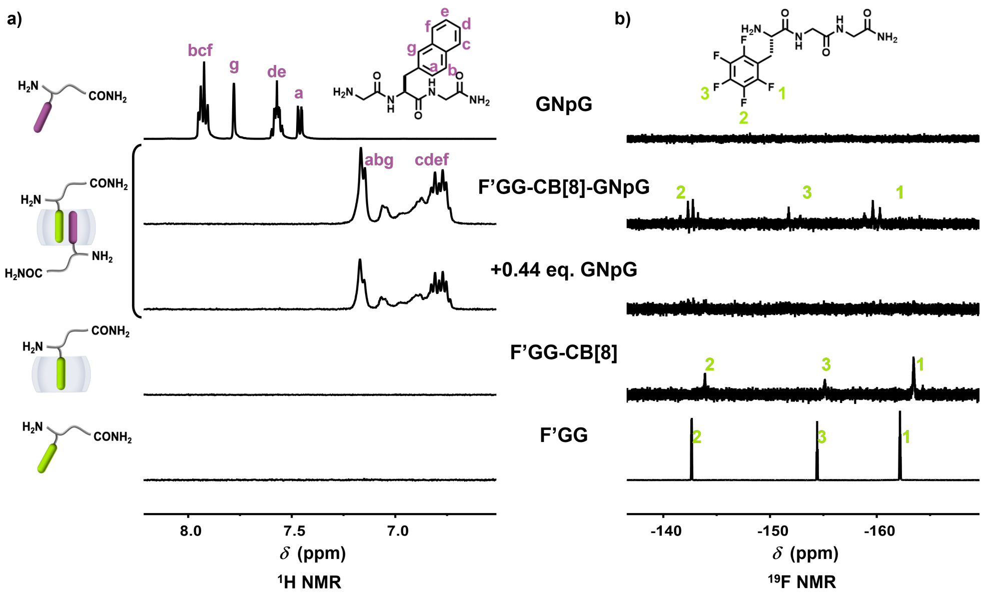

Figure S12: a) ^{1}H and b) ^{19}F NMR spectra obtained through titrations of GNpG to F'GG-CB[8] (1:1 complex) at the molar ratio 0.44:1.00 and 1.00:1.00 with three controls of free F'GG, pure F'GG-CB[8] complex and free GNpG. The ^{1}H NMR spectrum intensity of free GNpG was adjusted by a factor of 80 x and ^{19}F NMR spectrum intensity of free F'GG by 0.0002 x. (400 MHz, 298 K, D_2O, phosphate buffer, pH = 7.0)

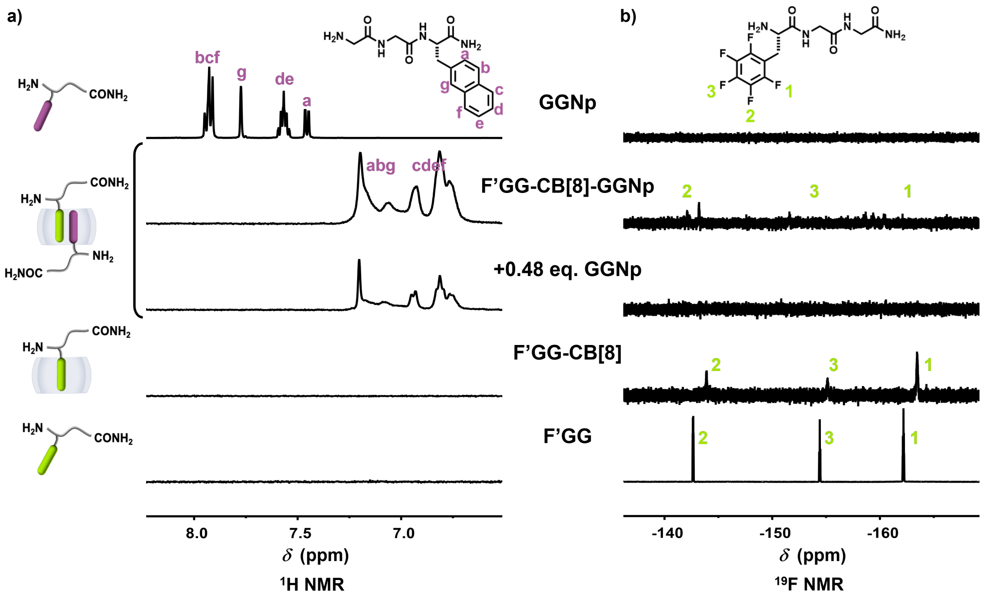

Figure S13: a) ^{1}H and b) ^{19}F NMR spectra obtained through titrations of GGNp to F'GG-CB[8] (1:1 complex) at the molar ratio 0.48:1.00 and 1.00:1.00 with three controls of free F'GG, pure F'GG-CB[8] complex and free GGNp. The ^{1}H NMR spectrum intensity of free GGNp was adjusted by a factor of 63 x and ^{19}F NMR spectrum intensity of free F'GG by 0.0002 x. (400 MHz, 298 K, D_2O, phosphate buffer, pH = 7.0)

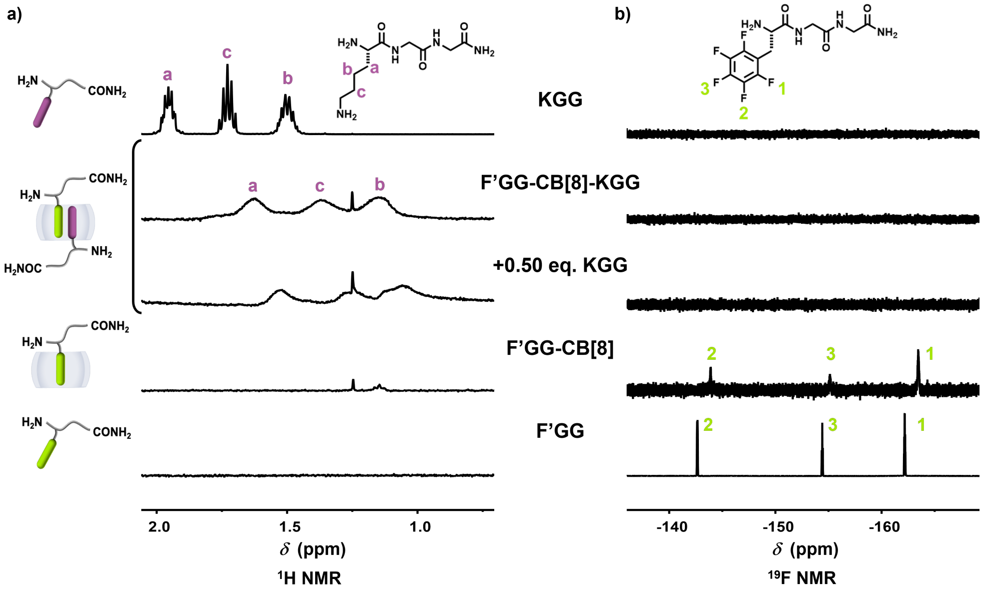

Figure S14: a) ^{1}H and b) ^{19}F NMR spectra obtained through titrations of KGG to F'GG-CB[8] (1:1 complex) at the molar ratio 0.50:1.00 and 1.00:1.00 with three controls of free F'GG, pure F'GG-CB[8] complex and free KGG. The ^{1}H NMR spectrum intensity of free KGG was adjusted by a factor of 17 x and ^{19}F NMR spectrum intensity of free F'GG by 0.0002 x. (400 MHz, 298 K, D_2O, phosphate buffer, pH = 7.0)

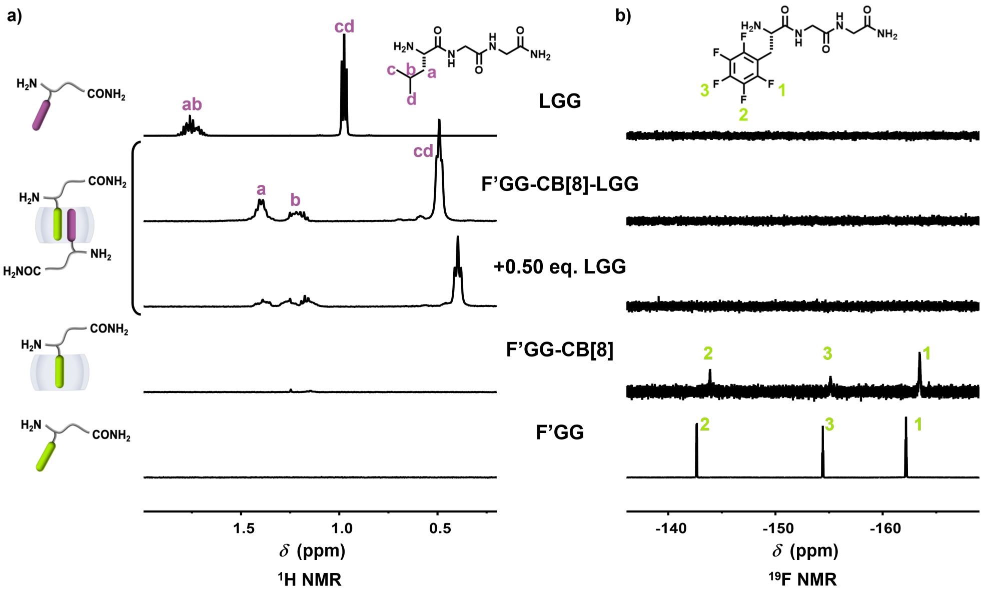

Figure S15: a) ^{1}H and b) ^{19}F NMR spectra obtained through titrations of LGG to F'GG-CB[8] (1:1 complex) at the molar ratio 0.50:1.00 and 1.00:1.00 with three controls of free F'GG, pure F'GG-CB[8] complex and free LGG. The ^{1}H NMR spectrum intensity of free LGG was adjusted by a factor of 24 x and ^{19}F NMR spectrum intensity of free F'GG by 0.0002 x. (400 MHz, 298 K, D_2O, phosphate buffer, pH = 7.0)

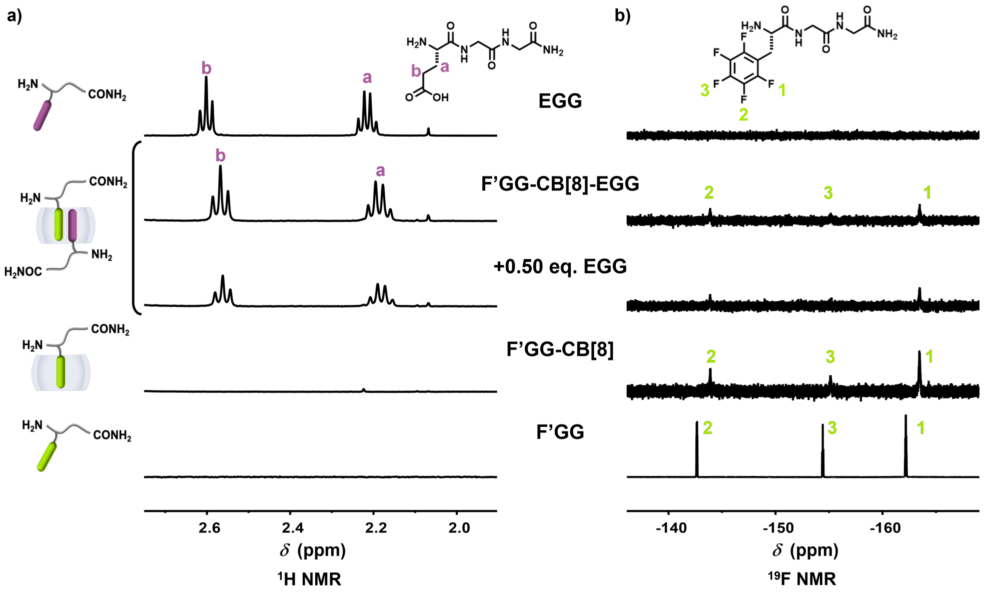

Figure S16: a) ^{1}H and b) ^{19}F NMR spectra obtained through titrations of EGG to F'GG-CB[8] (1:1 complex) at the molar ratio 0.50:1.00 and 1.00:1.00 with three controls of free F'GG, pure F'GG-CB[8] complex and free EGG. The ^{1}H NMR spectrum intensity of free EGG was adjusted by a factor of 25 x and ^{19}F NMR spectrum intensity of free F'GG by 0.0002 x. (400 MHz, 298 K, D_2O, phosphate buffer, pH = 7.0)

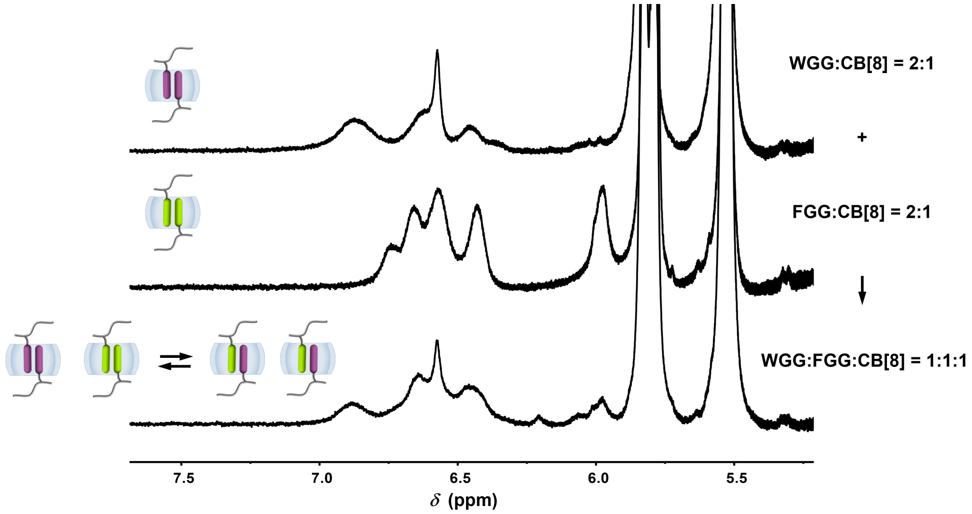

Figure S17: ^{1}H NMR of WGG-CB[8] (2:1 complex), FGG-CB[8] (2:1 complex) and equal molar mixture of those two complexes (WGG:FGG:CB[8] = 1:1:1). The spectrum intensity of FGG-CB[8] complex was adjusted by a factor of 1.3 x and spectrum intensity of WGG-CB[8], FGG-CB[8] mixture by 0.6 x. (400 MHz, 298 K, D_2O, phosphate buffer, pH = 7.0)

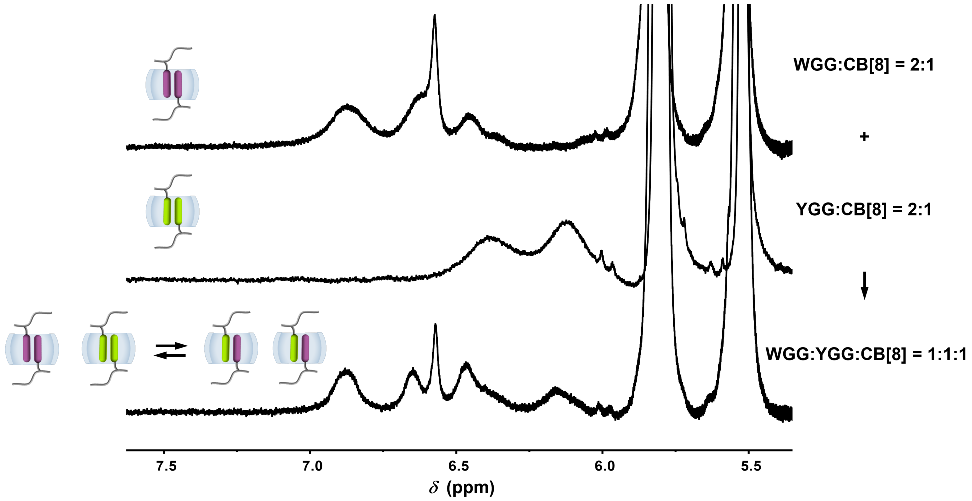

Figure S18: ^{1}H NMR of WGG-CB[8] (2:1 complex), YGG-CB[8] (2:1 complex) and equal molar mixture of those two complexes (WGG:YGG:CB[8] = 1:1:1). The spectrum intensity of YGG-CB[8] complex was adjusted by a factor of 1.2 x and spectrum intensity of WGG-CB[8], YGG-CB[8] mixture by 1.3 x. (400 MHz, 298 K, D_2O, phosphate buffer, pH = 7.0)

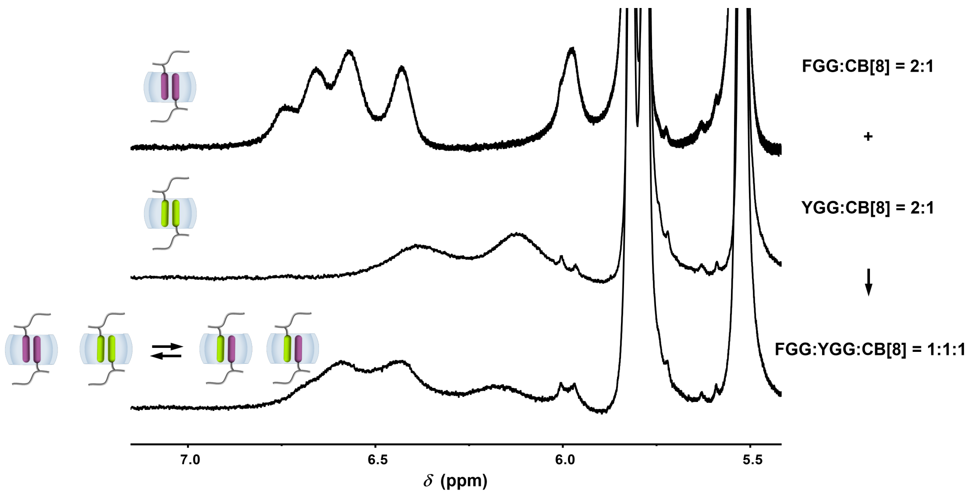

Figure S19: ^{1}H NMR of FGG-CB[8] (2:1 complex), YGG-CB[8] (2:1 complex) and equal molar mixture of those two complexes (FGG:YGG:CB[8] = 1:1:1). The spectrum intensity of YGG-CB[8] complex was adjusted by a factor of 0.9 x. (400 MHz, 298 K, D_2O, phosphate buffer, pH = 7.0)

As shown in Figure S17-19, tripeptide with N-terminal natural amino acid tryptophan (W), phenylalanine (F), tyrosine (Y) were able to form 2:1 homoternery complex with CB[8], respectively. However, when mixing any two of those homoternery complexes in equal molar ratio, due to their narcissistic self-sorting nature, an equilibrium of two homoternery complexes and one heteroternery complex exists in solution. For example, in Figure S17, WGG-CB[8] and FGG-CB[8] formed clear 2:1 complexes ranging from 6.9–6.5 ppm and 6.7–6.0 ppm, respectively. However, when mixed, instead of a new set of peaks indicating the formation of pure heteroternery complex, a wide range of peaks ranging from 6.9 to 6.0 ppm with similar peak shapes with both WGG-CB[8] and FGG-CB[8] complex appeared. This indicated a mixture of WGG-CB[8] 2:1, FGG-CB[8] 2:1, WGG-FGG-CB[8] 1:1:1 complexes that coexisted in the solution. The same argument can be applied to Figure S18 and S19. In contrast, in Figure S1, a clear new set of peaks appeared when F'GG, WGG, CB[8] were in equal molar which indicated a pure heteroternery complex of F'GG-WGG-CB[8] was formed. The above evidence further proves the use of unnatural amino acid pentafluorophenylalanine (F') is essential as sole natural amino acids-based system cannot achieve pure heterodimerization with CB[8].

4 Thermodynamic investigations by ITC

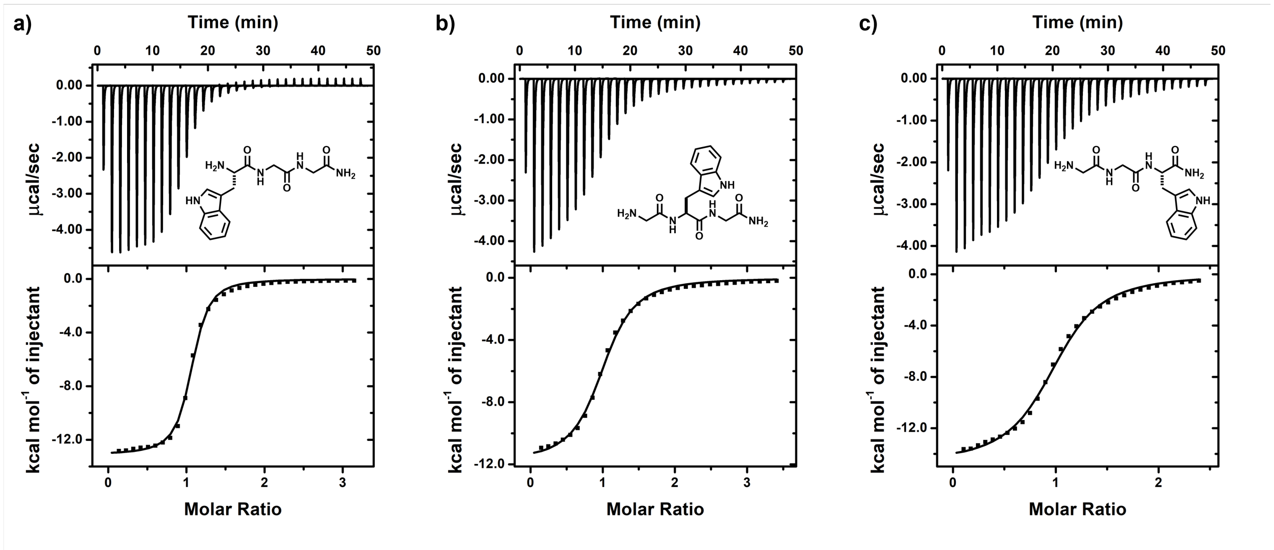

Figure S20: Representative ITC titration curves obtained by titrating a) WGG; b) GWG; c) GGW into F'GG-CB[8] (1:1 complex), respectively. All ITC experiments were repeated for 3 times. (298 K, 10 mM phosphate buffer, pH = 7.0)

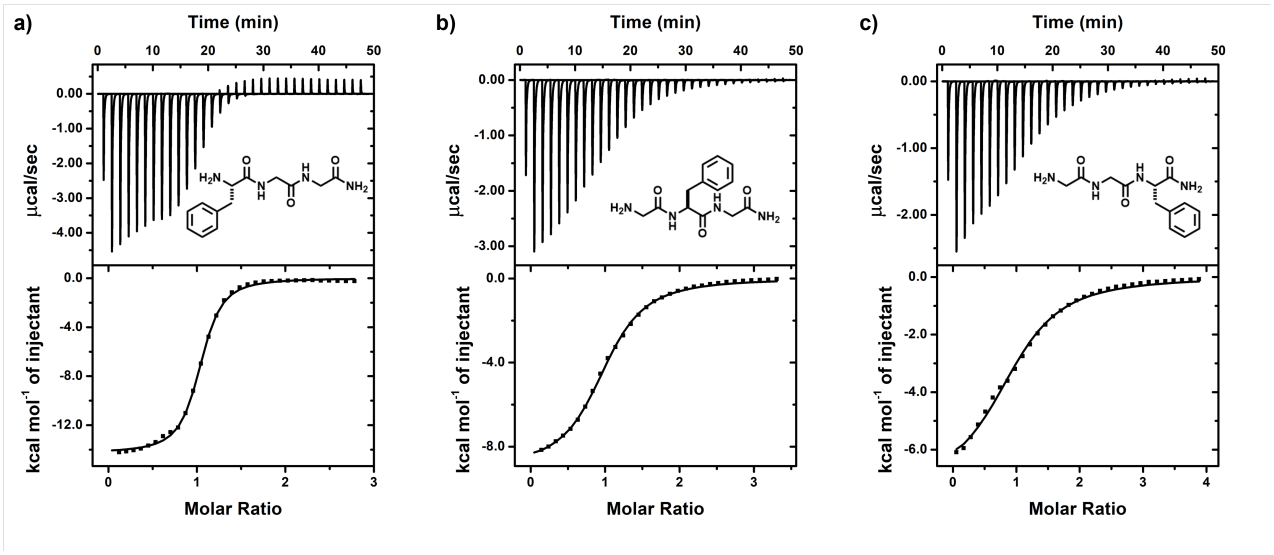

Figure S21: Representative ITC titration curves obtained by titrating a) FGG; b) GFG; c) GGF into F'GG-CB[8] (1:1 complex), respectively. All ITC experiments were repeated for 3 times. (298 K, 10 mM phosphate buffer, pH = 7.0)

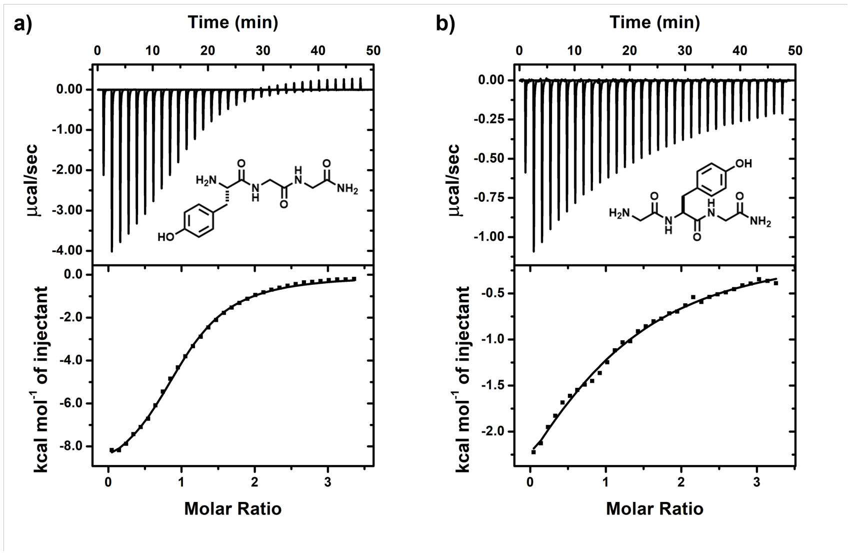

Figure S22: Representative ITC titration curves obtained by titrating a) YGG; b) GYG into F'GG-CB[8] (1:1 complex), respectively. All ITC experiments were repeated for 3 times. (298 K, 10 mM phosphate buffer, pH = 7.0)

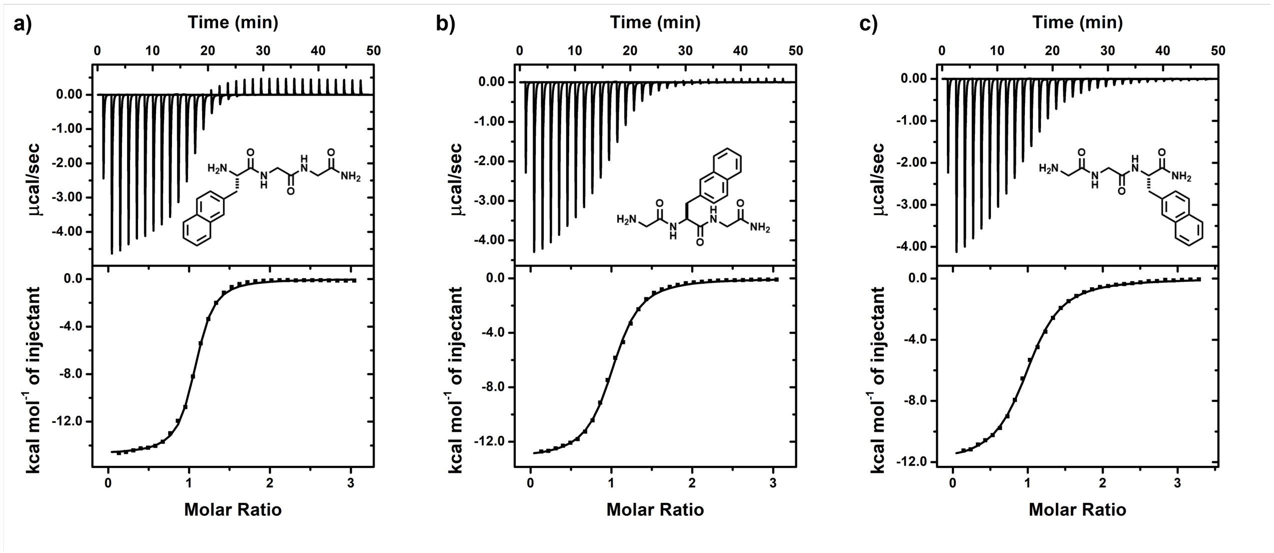

Figure S23: Representative ITC titration curves obtained by titrating a) NpGG; b) GNpG; c) GGNp into F'GG-CB[8] (1:1 complex), respectively. All ITC experiments were repeated for 3 times. (298 K, 10 mM phosphate buffer, pH = 7.0)

The complexation of GGY, KGG, LGG, EGG with F'GG-CB[8] were too weak (Ka < 10^2 M^{-1}) to be determined by ITC so that their non-binding curve (close to baseline) was not shown here.

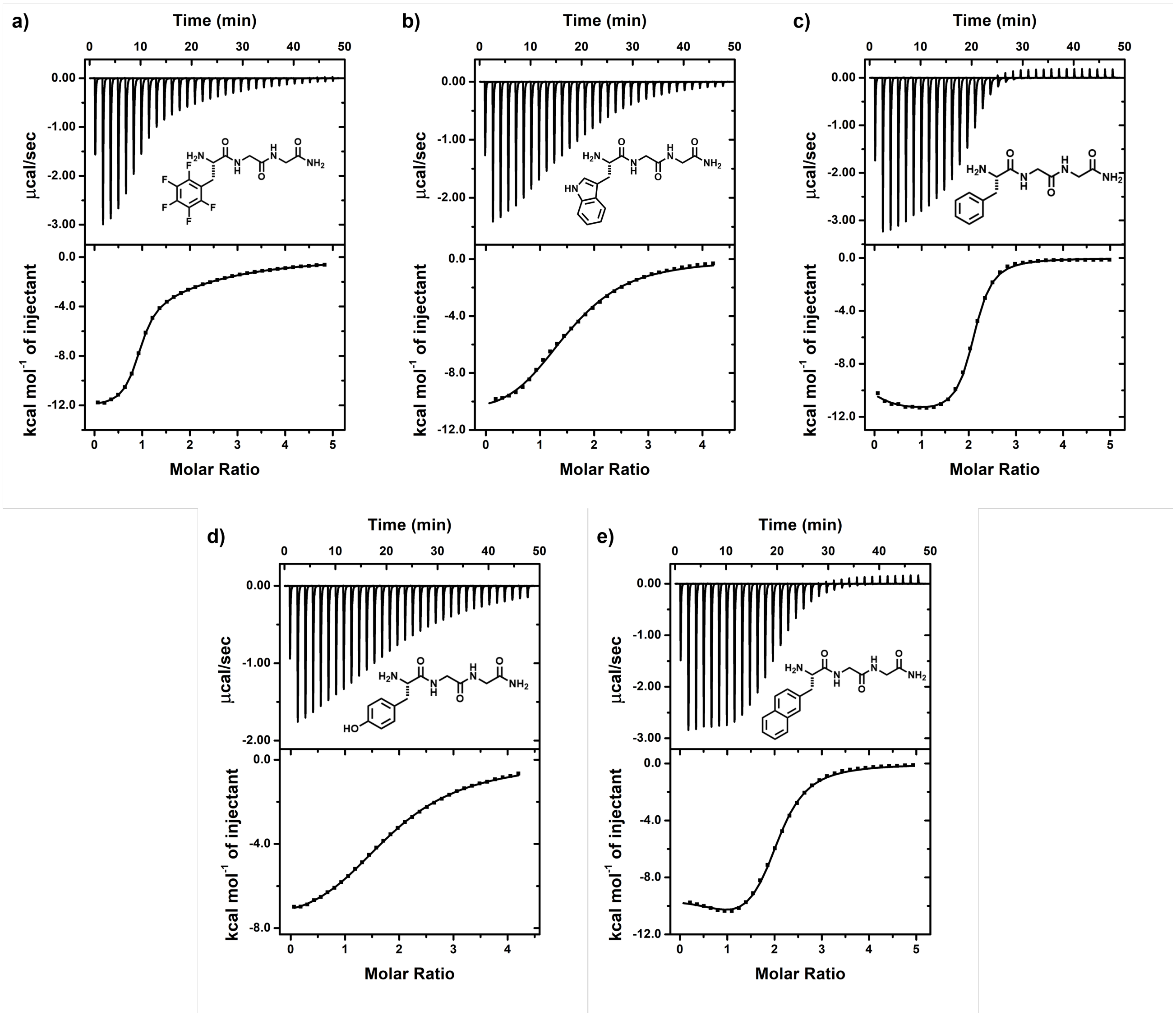

Figure S24: Representative ITC titration curves obtained by titrating a) F'GG; b) WGG; c) FGG; d) YGG; e) NpGG into CB[8], respectively. All ITC experiments were repeated for 3 times. (298 K, 10 mM phosphate buffer, pH = 7.0)

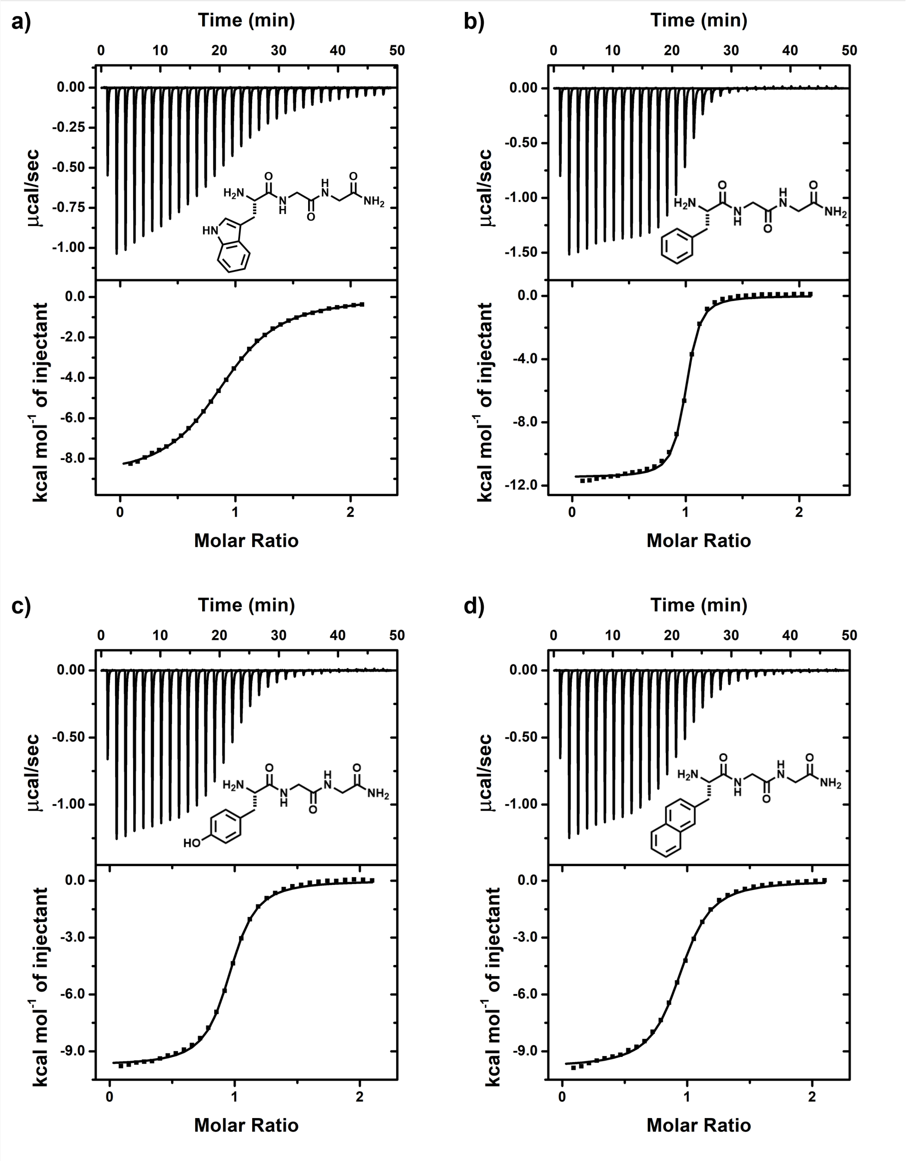

Figure S25: Representative ITC titration curves obtained by titrating a) WGG; b) FGG; c) YGG; d) NpGG into CB[7], respectively. All ITC experiments were repeated for 3 times. (298 K, 10 mM phosphate buffer, pH = 7.0)

Table S1: Thermodynamic data for homoternary complexation of aromatic tripeptides with CB[8][a]

model peptide	K_1 (10^4 M^{-1})	ΔH_1 (kcal mol^{-1})	-$T\Delta S_1$ (kcal mol^{-1})	K_2 (10^4 M^{-1})	ΔH_2 (kcal mol^{-1})	-$T\Delta S_2$ (kcal mol^{-1})	R[b]
F'GG	66 ± 4	-12.6 ± 0.4	4.5 ± 0.3	0.94 ± 0.09	-6.7 ± 0.5	1.3 ± 0.5	-
WGG	27 ± 5	-10.6 ± 0.1	3.2 ± 0.1	2.5 ± 0.1	-7.8 ± 0.5	1.8 ± 0.5	2291 ± 785
FGG	32 ± 3	-10.6 ± 0.1	3.1 ± 0.1	25 ± 1	-13.2 ± 0.1	5.8 ± 0.1	109 ± 27
YGG	8 ± 1	-7.9 ± 0.1	1.2 ± 0.1	1.1 ± 0.1	-9.5 ± 0.3	3.9 ± 0.3	70 ± 28
NpGG	66 ± 6	-9.8 ± 0.2	1.8 ± 0.2	7.8 ± 0.5	-12.5 ± 0.2	5.8 ± 0.2	199 ± 47

Note: [a]. All the above parameters were averaged with three replicates, and all error bars were calculated from standard deviation. [b]. The relative ratio of hetero- and homodimers shown as below:

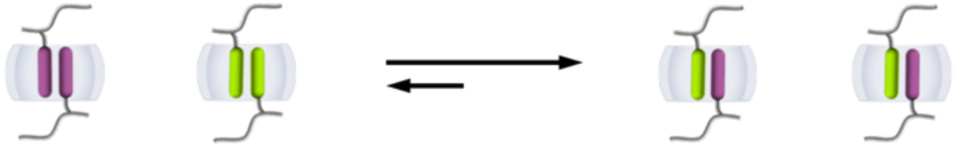

$$\mathbf{R} = \frac{[\text{heterodimers}]}{[\text{homodimers}]} = \frac{(K_{1(\mathrm{F'GG})} \cdot K_{a(\mathrm{XGG})})^2}{(K_1K_2)_{\mathrm{F'GG}} \cdot (K_1K_2)_{\mathrm{XGG}}}$$

Table S2: Thermodynamic data for binary complexation of aromatic tripeptides with CB[7][a]

model peptide	K (10^4 M^{-1})	ΔH (kcal mol^{-1})	-$T\Delta S$ (kcal mol^{-1})
WGG	13 ± 0.8	-8.91 ± 0.1	1.9 ± 0.1
FGG	280 ± 10	-11.4 ± 0.1	2.6 ± 0.1
YGG	82 ± 3	-9.56 ± 0.1	1.5 ± 0.1
NpGG	58 ± 3	-9.64 ± 0.2	1.8 ± 0.2

Note: [a]. All the above parameters were averaged with three replicates, and all error bars were calculated from standard deviation.

5 Statistical analysis on confocal imaging

Table S3: Summary on analysis of samples brightness by the mean grey value

	sample	mean grey value	resin beads diameter (μm)
A	resin	6.0 ± 0.4	173 ± 8
A'	resin-G_5F'	42.6 ± 11.1	207 ± 35
B	resin-G_5F' + WGG	45.0 ± 7.0	185 ± 20
B'	resin-G_5F' + WGG + CB[8]	35.8 ± 1.6	203 ± 16
B"	resin-G_5F' + WGG + CB[8] + DMADA	39.5 ± 6.9	229 ± 48
C	resin-G_5F' + WG_5-dansyl	57.9 ± 10.7	183 ± 16
C'	resin-G_5F' + WG_5-dansyl + CB[8]	85.4 ± 12.4	202 ± 16
C"	resin-G_5F' + WG_5-dansyl + CB[8] + DMADA	65.3 ± 8.6	204 ± 20

Statistical analysis was performed using Excel software with Daniel's XL Toolbox add-in. Significance between groups was determined by one-way analysis of variance (ANOVA) with Bonferroni-Holm post hoc test ($p < 0.05$), and error bars represent standard error of the mean.

Table S4: Statistical analysis of the samples' brightness

Group 1	Group 2	Significant?	Group 1	Group 2	Significant?
A	B'	Yes	B"	C	No
A	B	Yes	A'	C'	No
A	C	Yes	C	C'	No
A	C'	Yes	A'	C"	No
A	C"	Yes	C	C"	No
A	B"	Yes	A'	B'	No
B'	C'	Yes	A'	C	No
A	A'	Yes	B	B"	No
B'	C	Yes	C'	C"	No
B'	C"	Yes	B'	B"	No
B	C'	Yes	A'	B"	No
B	B'	Yes	A'	B	No
B"	C'	Yes			
B	C"	Yes			
B"	C"	Yes			
B	C	Yes			

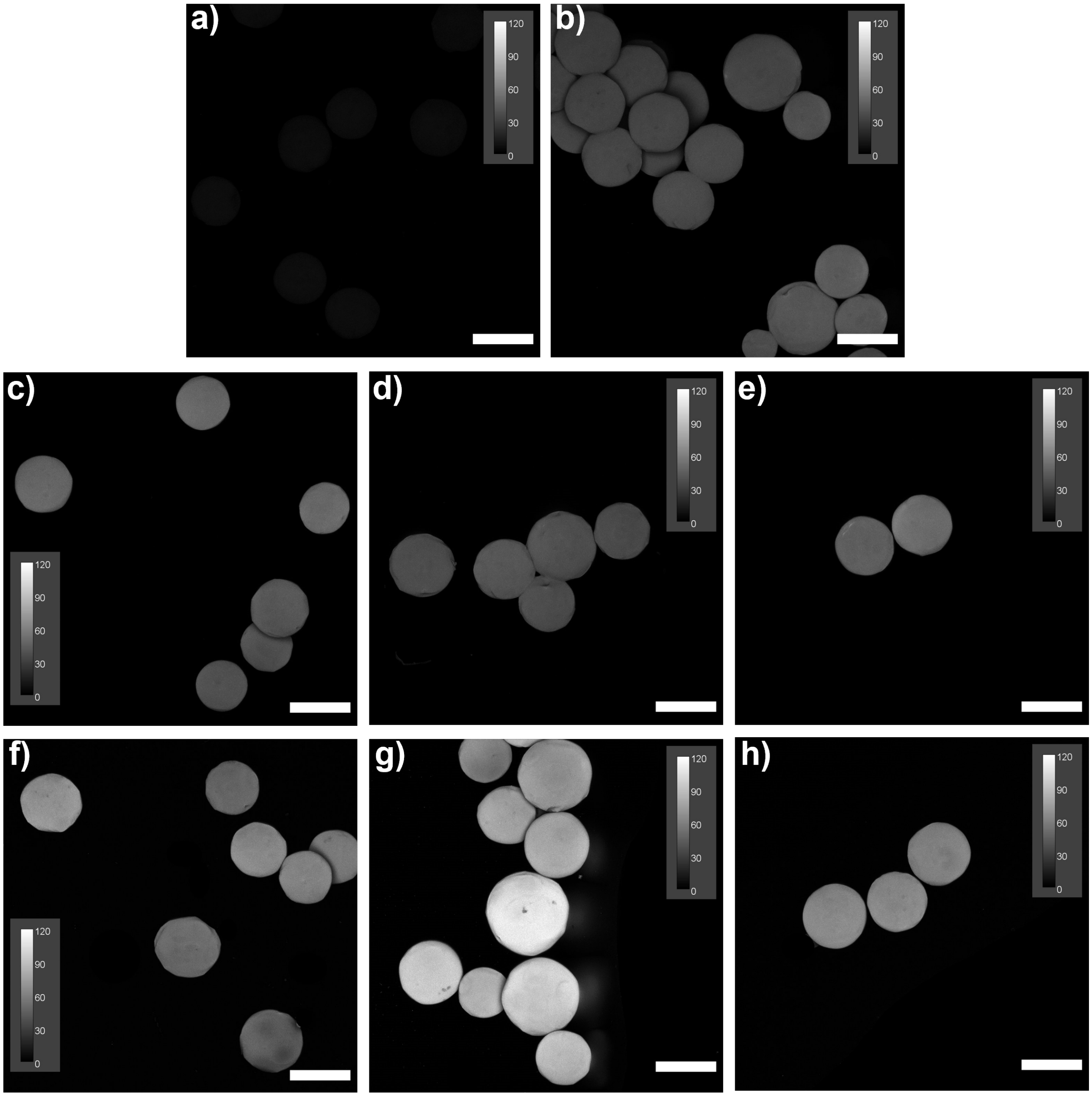

Figure S26: Confocal images of resin beads (10 mM). a) A - unmodified ChemMatrix resin; b) A' - F'GGGGG functionalized resin; c) B - F'GGGGG-resin with WGG; d) B' - F'GGGGG-resin with WGG and CB[8]; e) B" - F'GGGGG-resin with WGG, CB[8] and DMADA; f) C - F'GGGGG-resin with WGGGGG-dansyl; g) C' - F'GGGGG-resin with WGGGGG-dansyl and CB[8]; h) C" - F'GGGGG-resin with WGGGGG-dansyl, CB[8] and DMADA. Scale bars are 200 μm in all images.

6 Quantification of on-resin recognition by UV, ^{1}H NMR & CD

6.1 Quantitative analysis for on-resin recognition through UV measurements

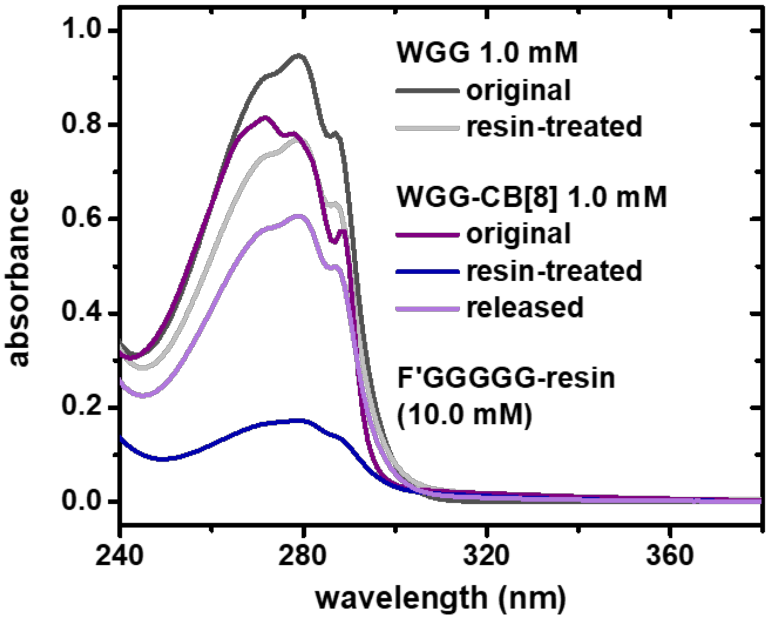

Figure S27: UV spectra for original (black) and resin-treated (grey) WGG, original (purple) and resin-treated (blue) WGG-CB[8] followed with competitive release (light purple) from F'GGGGG-resins by DMADA. All the solutions were prepared with 10 mM phosphate buffer of pH 7.0 in D_2O.

Table S5: UV absorbance at 279 nm for WGG and WGG-CB[8] at different states

	WGG		WGG-CB[8]		
state	original	resin-treated	original	resin-treated	released
repeat 1	0.9470	0.7695	0.7933	0.1805	0.6140
repeat 2	0.9473	0.7693	0.7938	0.1808	0.6143
repeat 3	0.9477	0.7690	0.7941	0.1808	0.6146
average	0.9474	0.7693	0.7937	0.1807	0.6143
std dev	0.0004	0.0003	0.0004	0.0002	0.0003
absorption efficiency	(0.9474 - 0.7693) / 0.9474 × 100% = 18.7%		(0.7937 - 0.1807) / 0.7937 × 100% = 77.2%		

6.2 Time-dependent investigations for on-resin recognition and release

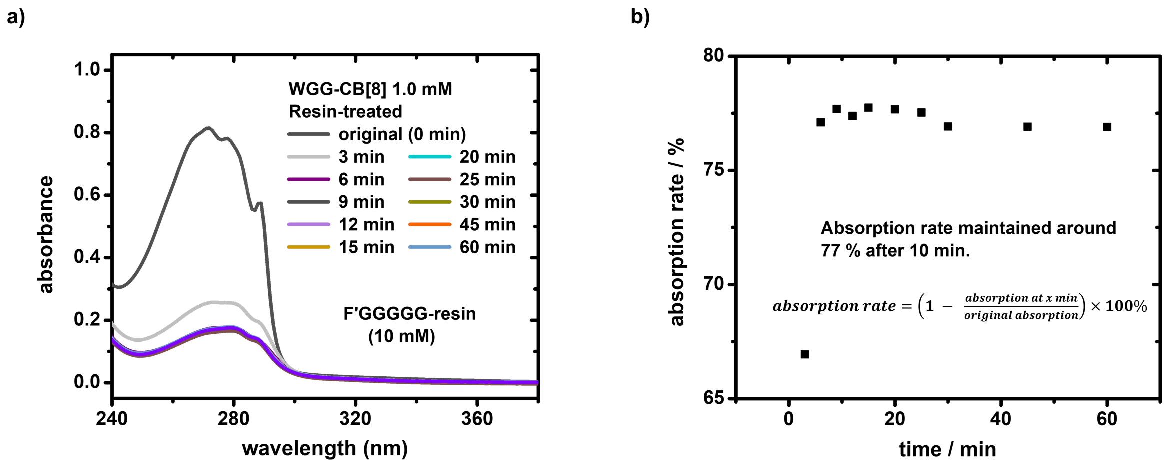

Figure S28: a) Time-dependent UV spectra of WGG-CB[8] treated with F'GGGGG-resins (3 – 60 min); b) plot and fitted curve for the absorption rate calculated upon UV absorbance at 279 nm (phosphate buffer, D_2O). Calculated using absorbance at 279 nm from Figure S28a, all samples were referenced to original absorbance to calculate the absorption rate.

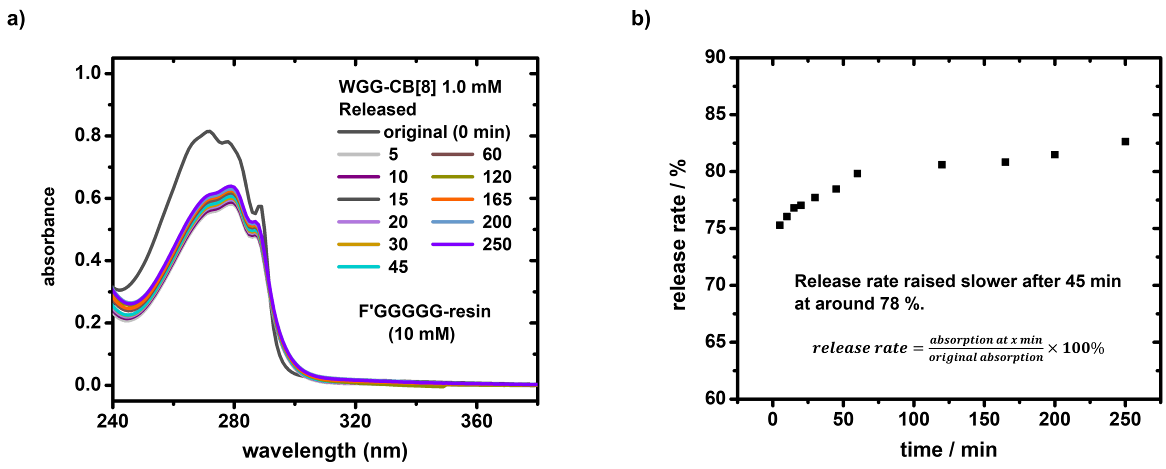

Figure S29: a) Time-dependent UV spectra of WGG-CB[8] released from F'GGGGG-resins (5 – 250 min); b) plot and fitted curve for the release rate curve calculated upon absorbance at 279 nm (phosphate buffer, D_2O). Calculated using absorbance at 279 nm from Figure S29a, all samples were referenced to original absorbance to calculate the release rate.

Shown in Figure S28, WGG-CB[8] bound to F'GGGGG-resin rapidly and the absorption rate plateaued around 77% after 10 min. In Figure S29, the release process occurred slower than binding, however, most of WGG was released after around 45 min at the plateau of 78%. 10 min and 45 min were chosen for on-resin recognition and release tests, respectively.

6.3 Multi-cycle on-resin recognition

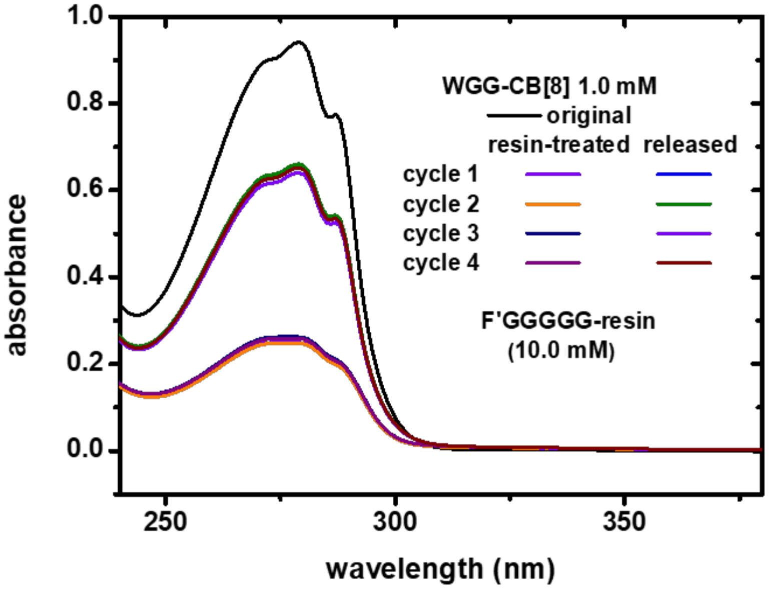

Figure S30: UV spectra of WGG-CB[8] (1.0 mM) before and after treated by F'GGGGG-resins (10 mM) for 4 consecutive cycles (phosphate buffer, D_2O).

Table S6: UV absorbance data at 279 nm for multi-cycle on-resin recognition[a]

	WGG-CB[8] original (Abs)	WGG-CB[8] resin-treated cycle 0 (Abs)	WGG-CB[8] resin-treated cycle 1 (Abs)	WGG-CB[8] resin-treated cycle 2 (Abs)	WGG-CB[8] resin-treated cycle 3 (Abs)
repeat 1	0.9383	0.2481	0.2465	0.2610	0.2572
repeat 2	0.9412	0.2499	0.2478	0.2626	0.2578
repeat 3	0.9431	0.2503	0.2500	0.2639	0.2605
average	0.9409	0.2494	0.2481	0.2625	0.2585
std dev	0.0024	0.0012	0.0018	0.0015	0.0018
efficiency (%)	-	(73.5 ± 0.3)%	(73.6 ± 0.3)%	(72.1 ± 0.4)%	(72.5 ± 0.4)%
recycling efficiency (%)	-	100.0%	(100.1 ± 0.6)%	(98.1 ± 0.7)%	(98.6 ± 0.7)%

Note: [a]. The efficiency results for Figure 4c were calculated from Table S6 using the following equations:

$$efficiency_{\text{on-resin recognition}} = 1 - \frac{abs_{\text{WGG-CB[8] resin-treated}}}{abs_{\text{WGG-CB[8] original}}}$$

$$recycling\ efficiency_{\text{cycle x}} = \frac{efficiency_{\text{cycle x}}}{efficiency_{\text{cycle 0}}}$$

6.4 On-resin selective isolation of aromatic peptides

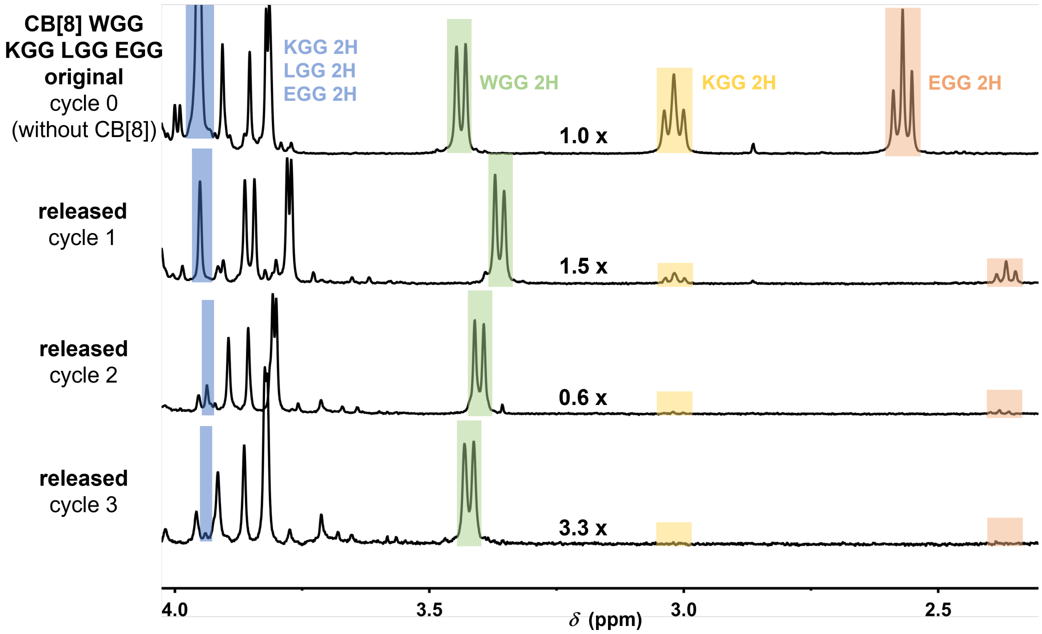

Figure S31: ^{1}H NMR of on-resin selective isolation of aromatic peptides from mixed peptide mixture (WGG, KGG, EGG, LGG 1.0 mM, phosphate buffer, D_2O) over 3 cycles using F'GGGGG-resins (10 mM).

The release DMADA-CB[8] complex might have some weak interactions (*i.e.* electrostatic interactions, hydrogen bonds) with the remaining tripeptides, which could lead to some changes in chemical shifts after release. The yield of the selectively separated WGG for a single cycle was 77% (Figure S28b), so the final yield of WGG after 3 cycles of selectively separation was 77%×77%×77% = 45.6%.

Table S7: ^{1}H NMR integration analysis for on-resin recognition within a peptide mixture[a]

	WGG		KGG		EGG		KGG+LGG+EGG	LGG	
	area	percentage (%)	area	percentage (%)	area	percentage (%)	total area	area	percentage (%)
cycle 0	1000.0	24.5	919.6	22.6	1129.5	27.7	3078.1	1029.0	25.2
cycle 1	1000.0	72.7	108.8	7.9	175.3	12.7	376.1	92.0	6.7
cycle 2	1000.0	86.7	25.6	2.2	40.5	3.5	153.2	87.1	7.6
cycle 3	1000.0	95.0	16.4	1.6	25.4	2.4	52.7	10.9	1.0

Note: [a]. All the above results of percentages were shown in Figure 4d.

6.5 On-resin recognition on GGGGGG resin

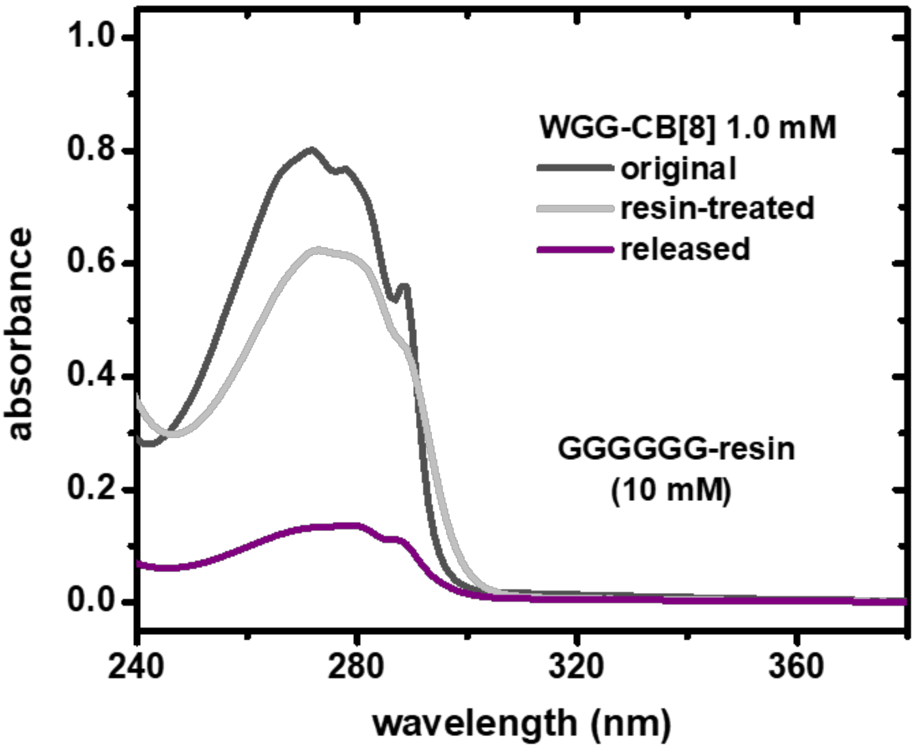

Figure S32: UV spectra of WGG-CB[8] (1.0 mM) before, after treated by and released from GGGGGG-resins (10 mM) (phosphate buffer, D_2O).

As shown in Figure S32, by using the same ChemMatrix resin but with different peptide GGGGGG other than F'GGGGG loaded, WGG-CB[8] complexes were almost not absorbed to the resin. The minimal amount of released signal may come from non-specific absorption. This confirmed that the absorption of WGG-CB[8] to F'GGGGG-resins was achieved by host-enhanced polar-π interactions.

6.6 On-resin recognition of insulin

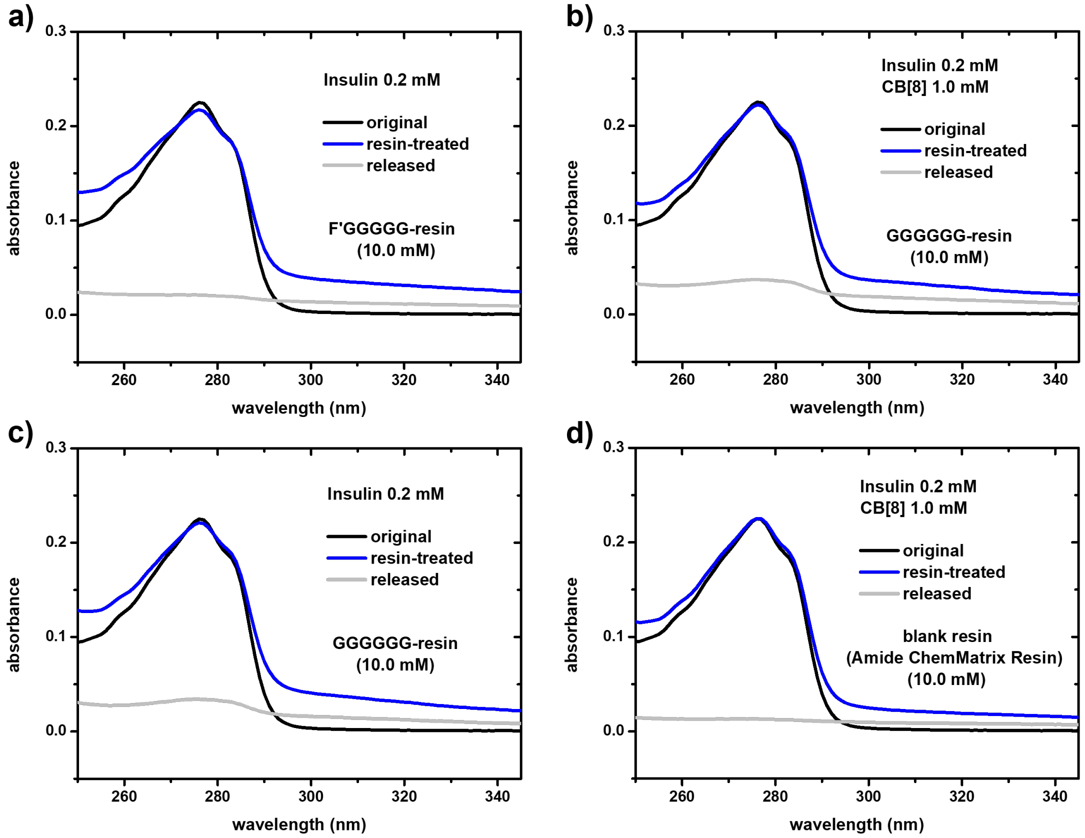

Figure S33: UV spectra of control experiments of insulin (0.2 mM) on-resin recognition. a) insulin (0.2 mM) on-resin recognition on F'GGGGG-resins (10 mM); b) insulin (0.2 mM) with CB[8] (1.0 mM) on-resin recognition on GGGGGG-resins (10 mM); c) insulin (0.2 mM) on-resin recognition on GGGGGG-resins (10 mM); d) insulin (0.2 mM) with CB[8] (1.0 mM) on-resin recognition on blank resins (H-Rink Amide ChemMatrix resin) (phosphate buffer, H_2O).

As shown in Figure S33a, under the same experimental conditions as Figure 4f without using CB[8], insulin showed minimal absorption to F'GGGGG-resin. In Figure S33b,c, by using the same ChemMatrix resin but with different peptide GGGGGG other than F'GGGGG loaded, regardless of the usage of CB[8], insulin exhibited negligible absorption in both cases. In Figure S33d, even insulin and CB[8] were used at the same time, there was almost no absorption to blank resin (H-Rink Amide ChemMatrix resin, as purchased, without any loaded peptides). This confirmed that the absorption of insulin-CB[8] to F'GGGGG-resins was achieved by host-enhanced polar-π interactions.

6.7 On-resin storage and on-demand release of insulin

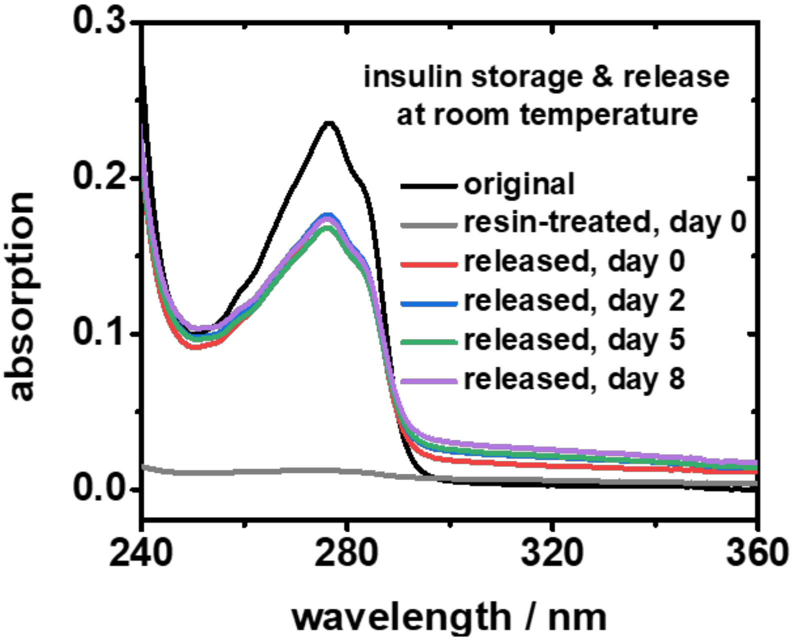

Figure S34: UV spectra of insulin (0.2 mM) solutions before and after treatment with F'GGGGG-CB[8]-resin (10.0 mM) followed by time-dependent release at day 0, 2, 5, & 8 (phosphate buffer, H_2O).

Table S8: UV absorbance data at 276 nm for on-resin storage and on-demand release of insulin on day 0, 2, 5, 8 of a 8-day experiment[a]

	insulin day 0 (abs)	insulin day 2 (abs)	insulin day 5 (abs)	insulin day 8 (abs)
original	0.23508	0.23508	0.23508	0.23508
resin-treated	0.01213	0.00845	0.01349	0.01162
released	0.17598	0.17676	0.16827	0.17394
absorption efficiency (%)	94.8%	96.4%	94.3%	95.1%
recycling efficiency (%)	100%	100.4%	95.6%	98.8%

Note: [a]. The efficiency results for Figure 4g were calculated from Table S8 using the following equations:

$$absorption\ efficiency_{\text{day x}} = 1 - \frac{abs_{\text{resin-treated, day x}}}{abs_{\text{original, day x}}}$$

$$recycling\ efficiency_{\text{day x}} = \frac{abs_{\text{released, day x}}}{abs_{\text{original, day x}}} \cdot \frac{abs_{\text{original, day 0}}}{abs_{\text{released, day 0}}}$$

Comparing to the 77% efficiency of treating F'GGGGG resin with WGG(1.0 mM) solution, a higher efficiency of 94% is observed when treating F'GGGGG resin with insulin(0.2 mM) solution. This phenomenon is actually one of the advantages of on-resin insulin stabilisation. Considering the Ka values of WGG and FGG are quite close, the difference of absorption efficiency between WGG and insulin is not simply related to the equilibrium calculations. Unlike WGG, insulin has two relatively long peptide chains consisting of 51 amino acids, thus its relatively large size could make it kinetically trapped into the crosslinked PEG(material of ChemMatrix resin) resin network. This can increase the absorption efficiency to about 94%, which is considerably higher than free small tripeptide WGG.

6.8 Secondary structure verification of released insulin

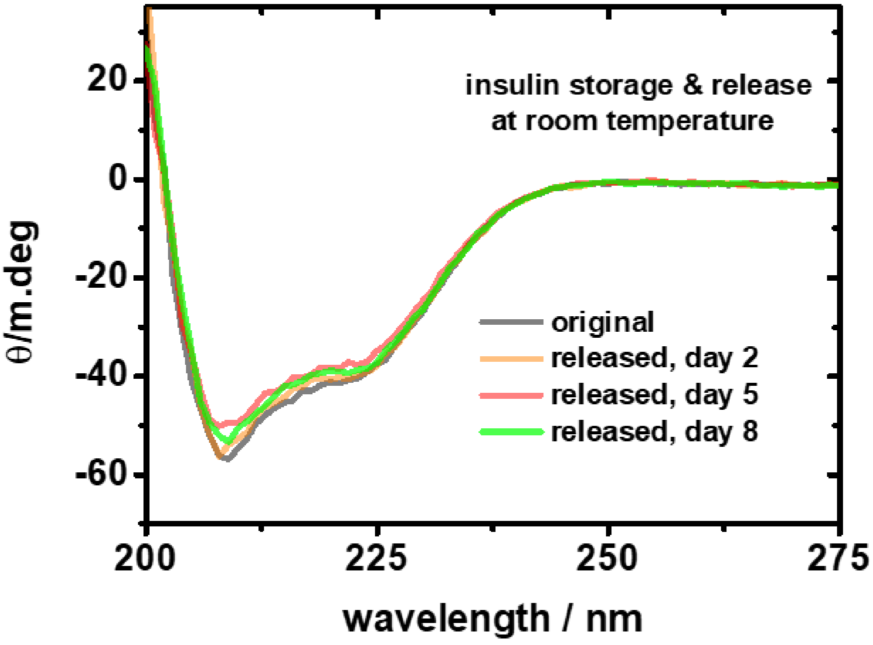

Figure S35: CD spectra of insulin solutions before and after treatment with F'GGGGG-CB[8]-resin followed by time-dependent release at day 2, 5, & 8 (phosphate buffer, H_2O).

Table S9: CD absorbance data at 208 nm for on-resin storage and on-demand release of insulin on day 2, 5, 8 of a 8-day experiment

	insulin, original (θm.deg)	insulin, day 2 (θm.deg)	insulin, day 5 (θm.deg)	insulin, day 8 (θm.deg)
repeat 1	-58.1	-58.1	-53.0	-52.4
repeat 2	-55.2	-55.4	-48.9	-52.3
repeat 3	-55.8	-55.7	-49.1	-52.2
signal	-56.4	-56.4	-50.3	-52.3
std dev	1.5	1.5	2.3	0.1
active ratio	-	100.0%	89.2%	92.7%

As shown in Figure S35 and Table S9, after 2, 5, 8 days of storage on functionalized resin, when released back into the solution phase, all three samples exhibited highly similar CD curves compared to freshly prepared insulin solution. This indicated that the secondary structures of insulin were intact after 2, 5, 8 days of storage under room temperature.

References

(S1) Kim, J.; Jung, I.-S.; Kim, S.-Y.; Lee, E.; Kang, J.-K.; Sakamoto, S.; Yamaguchi, K.; Kim, K. New cucurbituril homologues: syntheses, isolation, characterization, and X-ray crystal structures of cucurbit[n]uril (n= 5, 7, and 8). *Journal of the American Chemical Society* **2000**, *122*, 540–541.

(S2) Recipe: Insulin (0.5 mg/mL). Cold Spring Harbor Laboratory Press, 2014.
